# Stable isotope analysis confirms substantial changes in the fatty acid composition of bacteria treated with antimicrobial random peptide mixtures (RPMs)

**DOI:** 10.1038/s41598-022-13134-z

**Published:** 2022-07-04

**Authors:** Nina Wiedmaier-Czerny, Dorothee Schroth, Stephanie Krauß, Shiri Topman-Rakover, Aya Brill, Saul Burdman, Zvi Hayouka, Walter Vetter

**Affiliations:** 1 Department of Food Chemistry (170B), Institute of Food Chemistry, University of Hohenheim, 70593 Stuttgart, Germany; 2 Institute of Biochemistry, Food Science and Nutrition, The Robert H. Smith Faculty of Agriculture, Food and Environment, The Hebrew University of Jerusalem, Rehovot, 7610001 Israel; 3 Department of Plant Pathology and Microbiology, Institute of Environmental Sciences, The Robert H. Smith Faculty of Agriculture, Food and Environment, The Hebrew University of Jerusalem, Rehovot, 7610001 Israel

**Keywords:** Analytical chemistry, Lipids, Peptides

## Abstract

Resistance of plant-pathogenic bacteria to classic antibiotics has prompted the search for suitable alternative antimicrobial substances. One promising strategy could be the use of purposely synthesized random peptide mixtures (RPMs). Six plant-pathogenic bacteria were cultivated and treated with two RPMs previously found to show antimicrobial activity mainly by bacterial membrane disruption. Here, we show that bacteria treated with RPMs showed partly remarkable changes in the fatty acid pattern while those unaffected did not. Quantitative changes could be verified by compound specific isotope analysis of δ^13^C values (‰). This technique was employed due to the characteristic feature of stronger bonds between heavier isotopes in (bio)chemical reactions. As a proof of concept, the increase in abundance of a fatty acid group after RPM treatment was accompanied with a decrease in the ^13^C content and vice versa. We propose that our findings will help designing and synthesizing more selective antimicrobial peptides.

## Introduction

Since time immemorial, pathogenic bacteria have been a worldwide problem in the food sector^1,2^. Namely, these harmful bacteria can compromise agricultural yields^2^ and also cause food spoilage followed by health risks to consumers^3^. Hence, combating their occurrence by antibiotics is an important task. However, such efforts are getting increasingly catchier due to the resistance of pathogenic bacteria against classic antibiotics^1^. Therefore, one of the major challenges of our time is to track down alternative ways in combatting bacteria by active substances^4^.

One new and promising approach to substitute classic antibiotics could be the random antimicrobial peptide mixtures (RPMs)^5–7^. Synthesized dedicatedly in the laboratory, antimicrobial RPMs are similar in structure and they resemble the properties of naturally occurring antimicrobial peptides^5,6^, which are part of the immune response of animals and plants^7,8^. Conceptually, RPMs are produced from two purposefully selected amino acids. Namely, one amino acid has to be positively charged while the second amino acid should carry a hydrophobic residue^5^. Both features enable RPMs to attack the lipid bilayer of bacteria^5,6^. This antimicrobial peptide mixture has controlled chain length (20-mer) but the sequences are totally random^5^ (Fig. S1).

Despite notable antibacterial activity of RPMs with both different amino acids (including enantiomers), some of the results are difficult to predict and interpret^5–7^. For instance, some pathogenic bacteria were strongly affected while others showed no response to the RPMs and the mode of action remained unclear^9^. Arguably, RPMs may provoke stress on (the growth of) bacteria similarly to changes in temperature, or more basically, threats on lipid fluidity^10–12^. Under such pressure, bacteria but also higher organisms were shown to react by changes in the fatty acid (FA) composition^11,13^. In agreement with that, treatment of *Legionella pneumophila* with the antimicrobial peptide warnericin RK was followed by an increase in resistance which correlated with increasing shares of branched chain fatty acids (BCFAs) and a depletion of the average fatty acid chain length in the bacterial membranes^14^. Likewise, palmitic acid was more abundant in antibiotic-resistant variants of *Pseudomonas fluorescens* than in the ancestor strain^15^. Indications for changes in the fatty acids of bacteria in culture were also observed in a preliminary experiment in our laboratories with two pathogenic bacteria after treatment with RPMs.

The present study aimed to verify these indications by means of a cultivation experiment with six plant-pathogenic bacteria that are known to cause severe damage in agriculture (Table 1). The RPMs used in this study consisted of 20-mer peptide composed of either L-phenylalanine and L-lysine (FK_20_) or phenylalanine with D-lysine (FdK_20_) in 1:1 molar ratio and in random order (Fig. S1). Previous studies indicated an inactivation effect of FK_20_ on four of the bacterial samples (Table 1), while treatment with FdK_20_ had an inactivation effect on only three pathogenic bacteria (Table 1)^9^.

**Table 1:** Strains, abbreviations, agricultural threat of the plant-pathogenic bacteria used in this study and action of two random peptide mixtures.

Bacteria	Strain	Abbreviation (gram)	Action of FK_20_*	Action of FdK_20_*	Disease	Source
*Xanthomonas campestris* pv. *campestris*	ATCC 33,913	*X. campestris* (−)	+	+	Black rot disease of *Brassicaceae*	da Silva et al.^16^
*Xanthomonas perforans*	97-2	*X. perforans* (−)	+	+	Bacterial spot disease of tomato and pepper	Jones et al.^17^
*Acidovorax citrulli*	M6	*A. citrulli* (−)	−	−	Seedling blight or fruit rot and stains on the fruit	Burdman et al.^18^
*Pseudomonas syringae* pv. *tomato*	DC3000	*P. syringae* (−)	+	−	Bacterial spot disease of tomato	Cuppels^19^
*Clavibacter michiganensis*	NCPPB 382	*C. michiganensis* (+)	+	+	Bacterial canker and wilt of tomato	Meletzus and Eichenlaub^20^
*Streptomyces scabies*	Av	*S. scabies* (+)	−	−	Potato common scab	Topman et al.^9^

*(+) Effect, (−) no effect of the random peptide mixtures on the bacterial growth.

The six bacteria were cultivated to higher optical densities and aliquots were individually treated with the two RPMs. Effects of RPMs on bacterial growth was measured by means of the optical density at 600 nm (OD_600_)^9^. Then, lipids were extracted from freeze-dried samples, transesterified and the resulting fatty acid methyl esters (FAMEs) were analysed by gas chromatography coupled with mass spectrometry (GC/MS)^21,22^. Fatty acid patterns were related to those of the (untreated) control samples^23^. Evaluation was mainly based on four groups of FA, i.e. saturated (SFAs) and monoenoic ones (MUFAs), as well as *n-2*-methyl-branched or *iso*- (*i*FAs) and *n-3*-methyl-branched or *anteiso*-fatty acids (*a*FAs). Changes in the FA pattern were aimed to be verified by determination of stable carbon isotope ratios (δ^13^C values [‰]) of fatty acids as methyl esters by means of gas chromatography-combustion-isotope ratio mass spectrometry (GC-C-IRMS) according to Krauß and Vetter^24^. The rationale for involvement of GC-IRMS is that chemical bonds are stronger between heavy isotopes^24–26^. Accordingly, the remaining share of fatty acids that decrease in abundance must get enriched in ^13^C while fatty acids that increase in abundance will get lighter in carbon^24–26^. Hence, measuring such changes in the δ^13^C values (‰) of fatty acids are direct and independent proof of alterations in the fatty acid profile in response to RPMs.

## Materials and methods

### Solvents and chemicals

Nutrient broth (NB) was bought from Difco (Detroit, MI, USA). Methanol (99.85%), cyclohexane (99.5%) *n*-hexane (> 95%), and *iso*-propanol (99.8%) were ordered from Th. Geyer (Renningen, Germany). Sulfuric acid (96%) was bought from Carl Roth (Karlsruhe, Germany). Ethyl acetate (distilled, 99.5%), 3-hydroxyhexadecanoic acid (3-OH-16:0 (98%)), hydrocinnamic acid (3-phenylpropanoic acid, 3-Ph-3:0 (99%)), N,N-diisopropylcarbodiimide (DIC) and UltraPure water were purchased from Sigma Aldrich (Steinheim, Germany). N,N-Dimethylformamide (DMF) and piperidine were bought from Bio-Lab (Jerusalem, Israel). Undecanoic acid (> 97%) was ordered from Fluka (Steinheim, Germany). 8-Phenyloctanoic acid (8-Ph-8:0 (97%)) was bought from Alfa Aesar (Haverhill, MA, USA). 3-(Methylthio)propionic acid (3-MeS-3:0) was ordered from Santa Cruz Biotechnology (Dallas, TX, USA). Standards of *i*FAs and *a*FAs for identification were purchased from Larodan (Malmö, Sweden)^27^ while all other fatty acids were determined using the Supelco 37 component FAME mix (Sigma Aldrich, Steinheim, Germany) as reference standard^27^. The dicarboxylic FAME mix (di-7:0diME–di-12:0diME) was a standard mix isolated with countercurrent chromatography (CCC)^28^. The working gas CO_2_ (quality 4.5; δ^13^C value − 30.5‰) used for stable isotope analysis (IRMS), which was calibrated with the secondary reference material USGS40, was ordered from Westfalen company (Münster, Germany). The secondary reference material USGS40 (δ^13^C value (‰), − 26.39 ± 0.04‰ relative to Vienna Pee Dee Belemnite (V_PDB_)) was from the Reston Stable Isotope Laboratory (Reston, VA, USA)^24^. Tin capsules for liquids (3.5 × 5.5 mm, 0.04 mL volume, Sn purity 99.9%) were ordered from IVA Analysentechnik (Meerbusch, Germany).

### Synthesis of 20-mer random peptide mixtures (RPMs)

RPMs were synthesized using the method of Hayouka et al.^5^ In brief, RPMs were synthesized, with a Liberty Blue™ automated microwave peptide synthesizer (CEM Corp., Matthews, NC, USA), by standard Fmoc-based solid-phase peptide synthesis (SPPS) on Rink amide resin (0.6 mmol/g substitution, 0.1 mmol scale). N,N-Diisopropylcarbodiimide and OxymaPure (Chem-Impex INTL) dissolved in N,N-dimethylformamide (DMF) were used for activation steps, and 20% (v/v) piperidine in DMF was used for Fmoc-deprotection steps. Each coupling step was conducted with binary combinations of protected amino acids, with a stock solution containing a 1:1 ratio between L-phenylalanine (F) and L/D-lysine (K/dK) (2 equivalents from each amino acid, 0.2 mmol). After cleavage from the resin, peptides were lyophilized and kept at − 20 °C. Evaluation of molecular weight was done using MALDI-TOF (Fig. S2).

Compounds were solubilized in 100% UltraPure water in a concentration of 100 mg/mL and stored at − 20 °C. These active substances consisted of RPMs of 20 components FK and FdK.

### Bacterial samples, cultivation and treatment with RPMs

Fatty acid patterns of untreated strains of six bacteria (controls, Table 1) were recently studied by Wiedmaier-Czerny et al.^23^. In brief, cultivation was performed using existing standard protocols at the Institute of Biochemistry, Food Science and Nutrition (Hebrew University of Jerusalem, Israel)^9^. In brief, bacteria were grown in four 50 mL batches using nutrient broth medium (30 °C, with 180 rpm shaking) over night. Then, batches of the same bacteria were united, diluted to an OD_600_ of 0.1 in fresh nutrient broth medium (~ 10^8^ CFU/mL) and divided into different aliquots of 0.5 L each. To each bacterial suspension RPMs were added in approximately 0.5 × [Minimal inhibitory concentration] (MIC) except bacteria with MIC value of 200 µg/mL. For these bacteria the concentration was as maximum we have used in the assay (Table 2). In these conditions we were able to observe the damaged and the undamaged bacterial cells.

**Table 2:** Minimal inhibitory concentrations (MICs) (mean values and standard error) of FK_20_ and FdK_20_ towards tested bacteria after 24 h incubation (data from Topman et al.^9^), the used concentration of FK_20_ and FdK_20_ and the cultivated amount of control, FK_20_- and FdK_20_-treated bacteria samples.

	*S. scabies*	*A. citrulli*	*P. syringae*	*X. perforans*	*X. campestris*	*C. michiganensis*
MIC FK_20_ (µg/mL)	> 200	> 200	42 ± 6.8	35 ± 5.5	19 ± 3.3	9 ± 1.5
MIC FdK_20_ (µg/mL)	> 200	> 200	> 200	31 ± 5.4	25 ± 7.8	11 ± 1.3
FK_20_ used in the present study (µg/mL)	25	25	25	25	25	6
mg FK_20_/500 mL	12.5	12.5	12.5	12.5	12.5	3
FdK_20_ used in the present study (µg/mL)	25	25	25	12	12	6
mg FdK_20_/500 mL	12.5	12.5	12.5	6	6	3
Cultivated amount (acc. to OD_600_) of control (mg)	1061 (1799)*	291 (895)	810 (1333)	867 (1802)	1157 (1754)	1114 (2792)
Cultivated amount (acc. to OD_600_) of FK_20_ (mg)	2103 (1623)*	501 (1333)	313 (1634)	1082 (1491)	502 (1823)	643 (1150)
Cultivated amount (acc. to OD_600_) of FdK_20_ (mg)	782 (2095)*	606 (1264)	514 (1800)	1154 (1443)	1117 (1268)	971 (1590)

With *S. scabies* = *Streptomyces scabies, X. campestris* = *Xanthomonas campestris* pv. *campestris, X. perforans* = *Xanthomonas perforans, C. michiganensis* = *Clavibacter michiganensis, P. syringae* = *Pseudomonas syringae* pv. *tomato, A. citrulli* = *Acidovorax citrulli.*

*In brackets the cultivated amount of freeze-dried supernatant was shown.

MIC values determined by using sterile 96-well plates (Corning 3650) by a broth microdilution method in accordance to Hayouka et al.^5^ were adopted from Topman et al.^9^ were used to select appropriate RPMs concentrations for each bacterium. MICs (Table 2) were used in order to determine the RPMs concentrations for each bacterium in preparing samples for the fatty acid analysis.

Due to the larger amount of bacteria material required for further analysis, the amounts of RPMs were reduced in order to prevent complete standstill (Table 2). Evaluation was based by normalization of RPM-treated bacteria to % of controls (100%) as measured by OD_600_^9^. Namely, FK_20_ induced a reduction of the MIC of four bacteria (i.e. *X. campestris*, *X. perforans*, *C. michiganensis* and *P. syringae*) to between 4 and 21% of the corresponding untreated control samples (> 200 µg/mL) (Table 2). Two pathogenic bacteria, *S. scabies* and *A. citrulli*, were not affected by the treatment with FK_20_ (Table 2). Effects of FdK_20_ on the bacteria were very similar with the exception of *P. syringae* which was not affected by FdK_20_ (Table 2).

MICs (Table 2) were used in order to determine the RPM concentrations for each bacterium in preparing samples for the fatty acid analysis. Conditions of the previous MIC assay and the present treatments were different but main factors (i.e. RPM concentration, starting OD_600_ value, medium, incubation temperature) were the same. Hence, it can be assumed that the inhibition activity remained the same. After 24 h in a shaker (28 °C, 180 rpm), bacteria were centrifuged (15 min, 8000 rpm), washed 3 times with phosphate buffered saline (PBS) and followed by two additional washing steps with sterile double-distilled water. Then, bacteria were re-suspended in double-distilled water and freeze-dried before proceeding with lipid analysis.

### Transesterification of fatty acids from bacterial lipids

Fatty acids present in about 10 mg of freeze-dried bacteria (pellet) or about 100 mg of freeze-dried supernatant were converted into the corresponding methyl esters (FAMEs) according to Wendlinger et al.^22^ with slight modifications^23^. In short, 2 mL of 1% sulfuric acid in methanol was added and the samples were heated first for 90 min to 80 °C, then sonicated for 10 min and finally heated again for 30 min to 80 °C. Demineralized water (1 mL), aqueous saturated NaCl solution (1 mL) and *n*-hexane (2 mL) were added after cooling on ice. The hexane phase was transferred into a 1.5-mL amber glass vial after shaking and phase separation. The final concentration for measuring was ~ 70 µg FAMEs per mL *n*-hexane. Internal standard (ISTD) solution (10 µL of 100 µg/mL 11:0 ethyl ester (11:0-EE) for 1 mL, prepared according to Wiedmaier-Czerny et al.^23^) was added to each sample vial. Two initially measured samples (“first treatment”, samples labelled with _“*p*”_ in subscript, namely *X. campestris*_*p*_, *A. citrulli*_*p*_) were extracted by accelerated solvent extraction (ASE) by three different solvents^23^. These solvent systems in detail were 1.) 40 mL *n*-hexane/*iso*-propanol (3:2, *v/v*), 2.) 40 mL of the azeotropic mixture of cyclohexane/ethyl acetate (46:54, *w/w*) and 3.) 40 mL of the azeotropic mixture of methanol/ethyl acetate (44:56, *w/w*)^23^. The resulting extracts were transesterified and converted into sample solutions as shown above.

### Gas chromatography with mass spectrometry (GC/MS) analysis

FAME solutions were measured with a 5890 Series II Plus/5972 GC/MS system (system I) using the parameters of Wiedmaier-Czerny et al.^23^. Standard or sample solutions (1 µL) were injected in splitless mode onto a 60 m × 0.25 mm i.d. capillary column coated with Rtx-2330 (Restek, Bad Homburg, Germany). All samples were measured twice both in full scan mode (*m/z* 50–550) and in selected ion monitoring (SIM) mode (*m/z* 74, 79, 81, 87, 88 and 101) according to Thurnhofer et al.^21^. Individual FAMEs, the 37 K component FAME mix and an *iso*-/*anteiso*-FAME mix were used as reference standards^27,29^. In GC/MS-SIM mode, saturated FAMEs were determined with *m/z* 87 and monounsaturated FAMEs with *m/z* 74 using the ratio of both ions for verification^30^. Also hydroxy-FAMEs (OH-FAMEs) were detected in some of the samples^23^. However, since concentrations of OH-FAMEs were very low, they were not evaluated in this study.

Fatty acids are presented as contribution to the sum of FAMEs (= 100%) and were divided into four classes (*i*FAMEs, *a*FAMEs, saturated FAMEs and monoenoic FAMEs). In the following, control samples were indicated with “^C^”, samples treated with FK_20_ with “^FK^” and samples treated with FdK_20_ with “^FdK^” (all in superscript and placed in front of the corresponding value). In the following, fatty acids will be mostly denoted “FAs” although FAMEs were measured.

Uncommon FAs in the supernatant were quantified with reference standards ordered upon tentative identification by GC/MS. Dicarboxylic fatty acids were determined using the response factor of the shortest chain length present in the standard (Di7:0-diME) and quantified by *m/z* 74^28^. *S*-containing fatty acids were analysed relative to the standard 3-MeS-3:0. In GC/MS-SIM mode, 3-MeS-3:0-ME was determined with *m/z* 61. 3-Ph-FAMEs were determined with *m/z* 104 and 2-Ph-FAMEs with *m/z* 91. These ions (*m/z* 61, 91 and 104) were determined in an additional GC/MS-SIM run.

### Stable carbon isotope analysis (δ^13^C values (‰)) of FAMEs via gas chromatography-combustion-isotope ratio mass spectrometry (GC-C-IRMS)

Measurements were performed with a 6890 Series II GC (Agilent, Waldbronn, Germany) coupled via a ConfloIV interface to a Delta plus XP IRMS (Thermo Finnigan MAT, Bremen, Germany) system^31^ using the parameters of Krauß and Vetter^24^. A 1.5 m × 0.53 mm i.d. Rxi guard column (Restek, Bad Homburg, Germany) was linked by a SGE (Trajan) SilTite μ-union (0.32 − 0.53 mm; BGB Analytik, Rheinfelden, Germany) with a 30 m × 0.32 mm i.d. Optima 5MS column (Macherey–Nagel, Düren, Germany). The GC oven temperature was initially set for 1 min to 60 °C and then raised at 10 °C/min to 120 °C followed by 2 °C/min to 190 °C. After 1 min at this level, the temperature was raised with 20 °C/min to 250 °C (hold time 5 min). One microliter aliquots of each sample solution were injected.

GC-C-IRMS analyses were only performed with samples of the second treatment (*X. campestris*, *A. citrulli*, *P. syringae*, *S. scabies*, *X. perforans*, *C. michiganensis*) because available amounts of the first treatment (*X. campestris*_*p*_, *A. citrulli*_*p*_) were too low. For exact GC-C-IRMS measurements, FAs were anticipated to have concentrations of 20–40 ng/µL^24^. Due to the wide concentration ranges of individual FAs in the samples, bacterial FA solutions were stepwise diluted (Table S1) and 11:0-EE was added at 20 ng/µL as ISTD. The resulting dilutions were subsequently measured (1 µL injected, respectively) and δ^13^C values (‰) were determined for peaks which were in the anticipated concentration range, respectively. Each sample solution was analysed in triplicate.

#### Calculation mode for δ^13^C values (‰) of FAs and groups of FAs via GC-C-IRMS

Percent contributions of individual fatty acids to total fatty acids (%) were derived from conventional GC/MS (“Gas chromatography with mass spectrometry (GC/MS) analysis” section). Typically, all individual FAs contributing > 2% to the total fatty acids could be determined by GC-C-IRMS (labelled “B_i_” in Tables S2–S4). These represented 75–94% of the total fatty acids in the samples (ΣB_i_ (%), Tables S2–S4) (i.e. control as well as FK_20_- and FdK_20_-treated samples). The Σδ^13^C value (‰) of the FAs in a sample was calculated by summing up the measured individual δ^13^C values (‰) (δ^13^C_ind_) multiplied with the corresponding share (%) of the considered fatty acids followed extrapolation to 100% according to Eq. (1):

1 $$\Sigma \delta^{13} {\text{C}}\;\left( \textperthousand \right)\;{\text{value}} = \Sigma \left[ {{\text{A}}_{{\text{i}}} *{\text{B}}_{{\text{i}}} } \right]/\left[ {\Sigma {\text{B}}_{{\text{i}}} } \right]$$

with A_i_ being the measured δ^13^C value (‰) of an individual fatty acid (δ^13^C_ind_) and B_i_ being its contribution to the total fatty acids (%/100%) that could be measured followed by extrapolation to 100%.

Then, the δ^13^C_ind_ values (‰) of × FAs belonging to the same class of fatty acids (i.e. *i*FAs, *a*FAs, saturated (SFA) and monoenoic FAs (MUFA)) were summed up as exemplarily shown for *i*FAs in Eq. (2):

2 $$\Sigma\updelta ^{13} {\text{C}}_{{{\text{ind}}}} \left( {i{\text{FAs}}} \right)\left( \textperthousand \right) =\updelta ^{13} {\text{C}}_{{{\text{ind}}}} i{\text{FA-}}1 +\updelta ^{13} {\text{C}}_{{{\text{ind}}}} i{\text{FA-}}2 +\updelta ^{13} {\text{C}}_{{{\text{ind}}}} i{\text{FA-x}}.$$

Visualisation of relative changes between RPM-treated samples compared to the (untreated) control samples required a further normalization step. In a first step, the factor *f* between Σδ^13^C value (‰) of control (Σδ^13^C_cont_) of the sum of all fatty acid groups and Σδ^13^C value (‰) of treated samples (Σδ^13^C_treat_—either FK_20_ or FdK_20_) of the sum of all fatty acid groups (Tables S3, S4) was determined via Eq. (3):

3 $$f = \Sigma\updelta ^{13} {\text{C}}_{{{\text{cont}}}} /\Sigma\updelta ^{13} {\text{C}}_{{{\text{treat}}}}$$

Finally, Σδ^13^C_ind_ values (‰) of the four fatty acid groups (e.g. Σδ^13^C_ind_ (*i*FAs)) were normalized by multiplication with factor *f* (Eq. 4) (Tables S3, S4).

4 $$\Sigma\updelta ^{{{13}}} {\text{C}}_{{{\text{ind}},\;{\text{norm}}}} (\textperthousand) = \Sigma\updelta ^{{{13}}} {\text{C}}_{{{\text{ind}}}} \left( {i{\text{FAs}}} \right)*f$$

In the following the Σδ^13^C_ind, norm_ values (‰) of FK_20_- or FdK_20_-treated samples were compared to the Σδ^13^C_ind_ values (‰) of the control sample. For the sake of simplicity, the δ^13^C values (‰) were only described as δ^13^C_ind, norm_ values (‰).

Effects of the methylation of fatty acids on δ^13^C values (‰) were very small. Although MeOH was more depleted in ^13^C than the fatty acids (δ^13^C: − 43.2‰), this effect was diluted by the many unaffected carbons in the acyl chain which additionally were of very similar chain length. Initial tests with MUFAs in the control sample of *X. campestris* and in the FK_20_ treatment sample differed only by 0.1‰ (i.e. − 1.7‰ with MeOH correction and − 1.6‰ without MeOH correction). As a consequence, δ^13^C values (‰) of fatty acids were not corrected due to the methylation.

#### Bulk δ^13^C values (‰) by elemental analysis coupled to isotope ratio mass spectrometry (EA-IRMS)

Bulk δ^13^C values (‰) of (transesterified) FAs were determined with controls and FK_20_- and FdK_20_-treated samples of *X. campestris*, *X. campestris*_p_ and *X. perforans*. Aliquots of sample solutions corresponding with between 2 and 80 µg FAMEs were placed in tin capsules for liquids in triplicate (except *X. campestris*_p_ treated with FK_20_ due to insufficient sample material). The solvent was evaporated over a few minutes and capsules were sealed, weighed and introduced into the EA-IRMS system consisting of a Euro EA 3000 elemental analyser (Hekatech, Wegberg, Germany) and the IRMS system mentioned above using the parameters of Eibler et al.^31^. USGS40 (Reston Stable Isotope Laboratory, Reston, VA, USA) was used as secondary reference material. Ion currents *m/z* 44, *m/z* 45 and *m/z* 46 were determined relative to the working gas CO_2_ (δ^13^C value − 30.5‰) which was measured thrice per run for standardization^24^. Next to bulk δ^13^C values (‰) of FAs, some lyophilisates of control and FdK_20_-treated samples of *X. campestris* and *X. campestris*_p_ were also measured in triplicate by EA-IRMS. For this purpose, between 0.2 and 2.5 mg of lyophilisate were weighed into tin capsules for solids.

The resulting Σδ^13^C_ind, sum_ value (‰) of the sum of all FA groups was also compared with the bulk EA-IRMS values of the corresponding FA fraction. For example, the Σδ^13^C_ind, sum_ value (‰) in the *X. perforans* control sample was − 25.1‰ (Table 3) compared to − 24.3‰ obtained after EA-IRMS measurement (Table 3). The intrinsic differences between EA- and GC-C-IRMS data are in agreement with literature reports^24,25,31^. However, it was noticeable that the differences between EA-IRMS and GC-C-IRMS were larger when the percentage of FAs of the determined Σδ^13^C_ind_ values (‰) with GC-C-IRMS was lower (Table 3). Exemplarily, the EA bulk Σδ^13^C_EA_ values (‰) of *X. campestris* (control, FK_20-_- and FdK_20_-treated) and *X. perforans* (control, FK_20_ and FdK_20_ treated) were determined. Since these were almost identical to the bulk Σδ^13^C_ind_ values (‰) determined via GC-C-IRMS, it was assumed that the other bacteria would behave similarly.

**Table 3:** Bulk δ^13^C values (‰) of *X. campestris* and *X. perforans* measured with EA-IRMS and calculated after GC-C-IRMS measurements of the individual FAs.

	Σδ^13^C_EA_ (‰) (EA-IRMS)	Σδ^13^C_ind, sum_ (‰) (GC-C-IRMS)	% of FAME that were determined with GC-C-IRMS	δ^13^C EA value (‰) – δ^13^C GC value (‰)
***X. campestris***				
Control	− 22.3	− 20.4	83.6	− 1.9
FK_20_	− 22.4	− 21.3	84.9	− 1.1
FdK_20_	− 20.4	− 20.1	88.2	− 0.3
***X. perforans***				
Control	− 24.3	− 25.1	85.8	0.8
FK_20_	− 24.2	− 24.0	91.7	− 0.2
FdK_20_	− 26.3	− 26.4	94.3	0.1

### Quality control

Sample preparations and measurements of aliquots of freeze-dried materials of all main samples (*X. campestris*, *A. citrulli*, *P. syringae*, *S. scabies*, *X. perforans*, *C. michiganensis*) were carried out in duplicate (n = 2). The relative standard deviation between the duplicates was < 1.3%, with one exception (2.2% for *a*FAs of *X. campestris* in FdK_20_ sample 1). In the following, resulting mean values of % contribution of individual FAs and FA groups will be reported. Samples of the preliminary treatments (*X. campestris*_p_, *A. citrulli*_p_), prepared half a year earlier, were only analysed once. Previous analysis indicated that first and second cultivations of *X. campestris* and *A. citrulli* resulted in slightly different FA patterns^23^. Hence, these samples had to be treated independently.

Relative standard deviations of δ^13^C values (‰) determined by GC-C-IRMS in triplicate were generally < 3.8% and mostly < 1%. All EA-IRMS measurements in triplicate showed relative standard deviations of < 0.35%.

A thorough statistical analysis could not be carried out due to the mostly independent samples of this study. However, considering our hypothesis that changes of > 3% between RPM-treated and untreated control samples were significant was verified by means of a t-test (*p* < 0.05). Exceptions were *i*FAs and *a*FAs in FdK_20_ treated *S. scabies* (Δ ~ 2%, *p* = 0.04) and *a*FAs in FdK_20_ treated *C. michiganensis* (Δ = 0.1%, *p* = 0.01). As will be shown below, the strongest effect was observed for samples containing *i*FAs. Namely, when present *i*FAs were affected in 75% of the bacterial samples by more than 3% after treatment with FK_20_ (Fig. S3). By contrast, saturated FAs and *a*FAs were only affected by > 3% in 25–38% of bacterial samples, respectively.

## Results and discussion

### FAs of the supernatant of the centrifuged samples

In order to exclude that high shares of bacterial lipids were released from the bacterial biomass into the cultivated medium, the entire supernatant of the centrifuged samples was separated and analysed on FAMEs. In total, between ~ 70 and 2000 µg FAMEs were detected but only 6–265 µg FAMEs originated from the four FA groups detected in the untreated control samples (i.e. *i*FAs, *a*FAs, saturated and monoenoic FAs). This amount which represented < 1% compared to 20–76 mg FAMEs in the whole freeze-dried pellet was considered negligible. Vice versa, the supernatant featured several uncommon FAs which were not detected in the pellets. GC/MS analysis of methylated supernatant enabled to identify sulphur-containing FAs (dominated by 3-MeS-3:0, Fig. S4a), dicarboxylic FAs (succinic acid (Di4:0) and glutaric acid (Di5:0), Fig. S4b), and *n*-aromatic FAs (2-Ph-2:0, and 3-Ph-3:0, Fig. S4c). Since the growth medium featured amino acids, they were likely generated by the bacteria from methionine, aspartic acid and glutamic acid, and phenylalanine, respectively. However, the uncommon FAs occurred arbitrarily, and effects due to treatment with RPM could not be observed. Most importantly, the bacterial pellets contained virtually all of the four FA groups which will be studied.

### Effect of treatments with the RPM FK_20_ on the fatty acid pattern of the six bacteria samples

Possible effects of FK_20_ were evaluated by comparing the abundance of the four FA groups (*i*FAs, *a*FAs, saturated and monoenoic FAs) and partly individual FAs in pellets of centrifuged (untreated) controls and affected bacteria. With the exception of some very low abundant FAs, the same variety of FAs was detected in untreated controls and FK_20_-treated samples.

#### FA patterns of bacteria whose growth was unaffected by FK_20_ compared to untreated controls: definition of effects

*S. scabies* and *A. citrulli* were not affected in their growth by the treatment with FK_20_. In agreement with that the FA patterns of treated samples and the untreated control were virtually the same. In *S. scabies*, *i*FAs (^C^39.1% vs. ^FK^38.7%) and saturated FAs (^C^9.8% vs. ^FK^9.7%) were virtually the same in untreated control and treated sample (Fig. S5). Only the share of monoenoic FAs was slightly lower (^C^9.6% and ^FK^7.3%) in favour of *a*FAs (^C^41.5% and ^FK^44.5%). Likewise, the most prominent FAs (*a*15:0 (^C^26.1% vs. ^FK^27.5%), *i*16:0 (^C^22.2% vs. ^FK^20.6%) and *a*17:0 (^C^16.0% vs. ^FK^17.6%), Table S5) were barely affected by the treatment with FK_20_.

Also in *A. citrulli*, saturated FAs (^C^50.5% and ^FK^49.5%) and monoenoic FAs (^C^48.5% vs. ^FK^49.6%) were predominant and the slight differences were deemed irrelevant and considered as natural variations (Fig. S5, Table S6). In accordance, *p* values (> 0.05) did not indicate significant changes. As mentioned before, the control sample of the preliminary treatment (*A. citrulli*_p_) showed a slightly different fatty acid pattern (slightly richer in monoenoic FAs)^23^. However, the FA composition of *A. citrulli*_p_ was also almost the same before and after the treatment (^C^47.4% vs. ^FK^48.3% saturated FAs and ^C^52.5% vs. ^FK^51.6% monoenoic FAs) (Fig. S5).

Based on these samples, changes in the share of the four FA groups by less than 3% were considered to be subject to natural variations, while changes by > 3% in one FA group in RPM-treated bacteria will be treated as an effect caused by the treatment (“Quality control” section). This was taken into account when bacterial samples which were affected by FK_20_ were studied in the following.

#### FA patterns of bacteria whose growth was affected by FK_20_ compared to untreated controls: definition of effects

**Figure 1:**
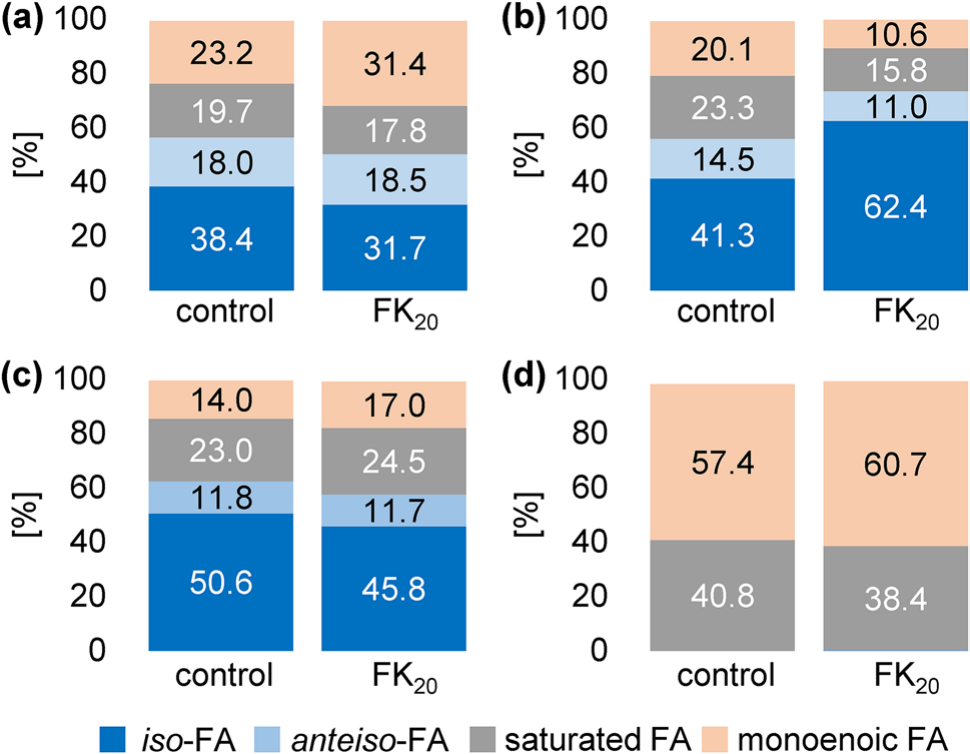
Percentage distribution of fatty acid groups (Σ*iso*-FAs, Σ*anteiso*-FAs, Σsaturated FAs and Σmonoenoic FAs) of (**a**) *Xanthomonas campestris* pathovar (pv) *campestris* (*X. campestris*), (**b**) *Xanthomonas perforans* (*X. perforans*), (**c**) *Clavibacter michiganensis* (*C. michiganensis*), and (**d**) *Pseudomonas syringae* pv. *tomato* (*P. syringae*) samples without treatment (control) and with FK_20_ treatment.

Remarkably enough, all four bacteria samples (*X. campestris, X. perforans, C. michiganensis* and *P. syringae*) whose growth was impaired by FK_20_ showed higher changes of > 3% in the abundance of at least one of the four FA groups (Fig. 1, Table 4).

**Table 4:** Differences in the percentage distribution (%) and the δ^13^C_ind, norm_ values (‰) of the fatty acid groups determined with GC-C-IRMS between the FK_20_- or FdK_20_- treated samples and the untreated control sample.

	FK_20_-control FAME (%)	FK_20_-control δ^13^C_ind, norm_ (‰)	FdK_20_-control FAME (%)	FdK_20_-control δ^13^C_ind, norm_ (‰)
***A. citrulli***				
SFA	− 1.0	0.2	− 1.0	0.3
MUFA	1.0	0.0	1.0	0.0
***S. scabies***				
*i*FA	− 1.6	0.4	− 2.2	0.4
*a*FA	2.1	− 0.4	1.9	− 0.3
SFA	− 0.4	0.0	0.3	− 0.1
***P. syringae***				
SFA	− 2.5	0.3	− 0.5	0.0
MUFA	2.5	− 0.3	0.5	0.0
***C. michiganensis***				
*i*FA	− **4.5**	0.8	0.4	− 0.5
*a*FA	0.4	− 0.1	− 0.4	0.4
SFA	**2.9**	− 0.6	0.0	0.0
MUFA	1.2	0.0	0.0	0.0
***X. campestris***				
*i*FA	− **6.5**	1.2	**9.6**	− 2.0
*a*FA	0.2	0.1	− 0.5	0.3
SFA	− **3.2**	0.6	− **15.1**	**2.9**
MUFA	**9.5**	− 1.9	**5.9**	− 1.3
***X. perforans***				
*i*FA	**21.2**	− **4.6**	**20.4**	− **4.5**
*a*FA	− **4.6**	1.0	− **5.6**	1.3
SFA	− **8.5**	2.1	− **4.9**	1.1
MUFA	− **8.2**	1.5	− **9.9**	2.1

Significant values are in [bold].

With *SFA* Saturated fatty acids, *MUFA* Monounsaturated fatty acids, *iFA iso*-fatty acids, *aFA anteiso*-fatty acids.

Treatment of *X. campestris* with FK_20_ reduced the share of *i*FAs by ~ 20% compared to the control (^C^38.4% → ^FK^31.6%, Fig. 1a). Also, the share of saturated FAs was slightly decreased from ^C^19.7% to ^FK^17.8% (Fig. 1a). This decline was compensated for by a strong increase of monoenoic FAs by 8.2% from ^C^23.2% to ^FK^31.4% (Fig. 1a). Only the share of *a*FAs remained nearly the same (Fig. 1a). The observed changes were almost entirely due to a decrease in *i*15:0 by 5% (^C^23.7% → ^FK^18.8%) in favour of 16:1*n-7* which almost doubled its share (^C^11.1% → ^FK^20.7%) (Table S7). Still, the same variety of FAs was detected before and after FK_20_ treatment (Table S7). Although the FA pattern of the control sample of *X. campestris* and the preliminary treatment *X. campestris*_p_ differed slightly, relative changes in *X. campestris*_p_ induced by the treatment with FK_20_ were on a similar level as in *X. campestris* (Fig. S6). Hence, these measurements produced strong evidence that the observed changes were meaningful.

In accordance with that, FK_20_ treatment of *X. perforans* also caused a remarkable shift in the FA pattern compared to the untreated control sample. Specifically, the enormous increase of *i*FAs by more than 20% (^C^41.3% → ^FK^62.4%, Fig. 1b) was compensated for by notable decreases of the abundance in monoenoic FAs (^C^20.1% → ^FK^10.6%) > saturated FAs (^C^23.3% → ^FK^15.8%) > *a*FAs (^C^14.5% → ^FK^11.0%) (Fig. 1b). Both *i*15:0 (^C^19.1% → ^FK^27.2%) and *i*17:0 (^C^10.3% → ^FK^16.6%) similarly increased in relative abundance, while great reduction was observed for 16:0 (^C^14.8% → ^FK^9.7%), 16:1*n-7* (^C^13.8% → ^FK^6.3%) and *a*15:0 (^C^11.2% → ^FK^7.6%) (Table S8).

Treatment of *C. michiganensis* with FK_20_ also effected the relative abundance of *i*FAs but in the opposite way as found above for *X. perforans*. Namely, FK_20_ treatment of *C. michiganensis* caused a decrease in *i*FAs (^C^50.6% → ^FK^45.8%) (Fig. 1c) in favour of slight increases of saturated (^C^23.0% vs. ^FK^24.5%) and monoenoic FAs (^C^14.0% vs. ^FK^17.0%, Fig. 1c). Only the share of *a*FAs remained the same (^C^11.8% → ^FK^11.7%, Fig. 1c). Changes in *i*FAs were mainly due a decrease in *i*15:0 (^C^37.8% → ^FK^33.1%) (Table S9).

*P. syringae* only featured saturated FAs (which decreased from ^C^41.0% to ^FK^38.4%) and monoenoic FAs (which increased from ^C^57.7% to ^FK^60.7%) (Fig. 1d). Assumedly, the lower magnitude of changes in the FA pattern compared to *X. campestris*, *X. perforans*, and *C. michiganensis* was mainly due to the absence of *i*FAs in *P. syringae* (Table S10). Second, decreasing MIC values in the order *P. syringae* > *X. perforans* > *X. campestris* > *C. michiganensis* also indicated the least vulnerability of this bacterium (Table 2). Although changes in FAs of *P. syringae* caused by the treatment of FK_20_ were comparably small, they still exceeded those observed with *A. citrulli* whose growth was not affected by FK_20_.

Despite deviations in amount and response to MIC values, all examples indicated that changes in their FA pattern could serve as indicators for bacterial response on RPMs. For further support of this hypothesis, the experiments were repeated with another RPM, FdK_20_ with the only difference between them beeing the stereocenter of the cationic amino acid (lysine).

### Effect of treatments with the RPM FdK_20_ on the fatty acid pattern of the six bacteria samples

FdK_20_ treatment had no effect on the bacterial growth of three bacteria (*S. scabies*, both *A. citrulli* and *A. citrulli*_p_, as well as *P. syringae*) also the FA patterns were not changed compared to the corresponding controls (Fig. S5). Differently to FK_20_ (“Effect of treatments with the RPM FK_20_ on the fatty acid pattern of the six bacteria samples” section), FdK_20_ did not impair the growth of *P. syringae* (Table 2). However, the absence of response to *P. syringae* was in agreement with no changes in FA composition.

Growth of the remaining three bacteria was similarly affected by FdK_20_ as by FK_20_. MIC values were slightly different but still in the order *X. perforans* > *X. campestris* > *C. michiganensis* (Table 2). Also, FdK_20_ induced similar changes in the FA patterns of *X. perforans* as FK_20_ (Table 5) but effects of FdK_20_ on *X. campestris* and *C. michiganensis* were different to FK_20_.

**Table 5:** Percentage distribution of fatty acid groups of all six plant-pathogenic bacteria and contribution to Σδ^13^C_ind, norm_ values (‰) obtained from GC-C-IRMS of individual FAs and Σδ^13^C_ind, norm_ values (‰) of control and FK_20_-/FdK_20_-treated samples.

FA groups	Control		FK_20_		FdK_20_	
	%	Σδ^13^C_ind, norm _(‰)	%	Σδ^13^C_ind, norm_ (‰)	%	Σδ^13^C_ind, norm_ (‰)
***A. citrulli***						
SFA	48	− 10.8	47	− 10.6	47	− 10.5
MUFA	52	− 11.6	53	− 11.8	53	− 11.8
Sum	100	− 22.4	100	− 22.4	100	− 22.4
***S. scabies***						
*i*FA	44	− 10.2	43	− 9.8	42	− 9.8
*a*FA	47	− 11.0	49	− 11.4	49	− 11.3
SFA	9	− 1.9	8	− 1.9	9	− 2.0
Sum	100	− 23.1	100	− 23.1	100	− 23.1
***P. syringae***						
SFA	37	− 6.4	35	− 6.1	37	− 6.4
MUFA	63	− 10.2	65	− 10.5	63	− 10.2
Sum	100	− 16.6	100	− 16.6	100	− 16.6
***C. michiganensis***						
*i*FA	55	− 12.3	50	− 11.5	55	− 12.8
*a*FA	15	− 4.1	15	− 4.2	14	− 3.7
SFA	26	− 5.9	29	− 6.5	26	− 5.9
MUFA	5	− 1.1	6	− 1.1	5	− 1.1
Sum	100	− 23.4	100	− 23.4	100	− 23.4
***X. campestris***						
*i*FA	39	− 8.3	33	− 7.1	49	− 10.3
aFA	20	− 4.3	20	− 4.2	19	− 4.0
SFA	21	− 4.1	18	− 3.5	6	− 1.2
MUFA	19	− 3.6	29	− 5.5	25	− 4.9
Sum	100	− 20.4	100	− 20.4	100	− 20.4
***X. perforans***						
*i*FA	45	− 12.0	66	− 16.6	65	− 16.5
*a*FA	15	− 3.9	11	− 2.9	10	− 2.6
SFA	24	− 5.9	15	− 3.8	19	− 4.8
MUFA	16	− 3.3	8	− 1.8	6	− 1.2
Sum	100	− 25.1	100	− 25.1	100	− 25.1

With *SFA* Saturated fatty acids, *MUFA* Monounsaturated fatty acids, *iFA iso*-fatty acids, *aFA anteiso*-fatty acids.

**Figure 2:**
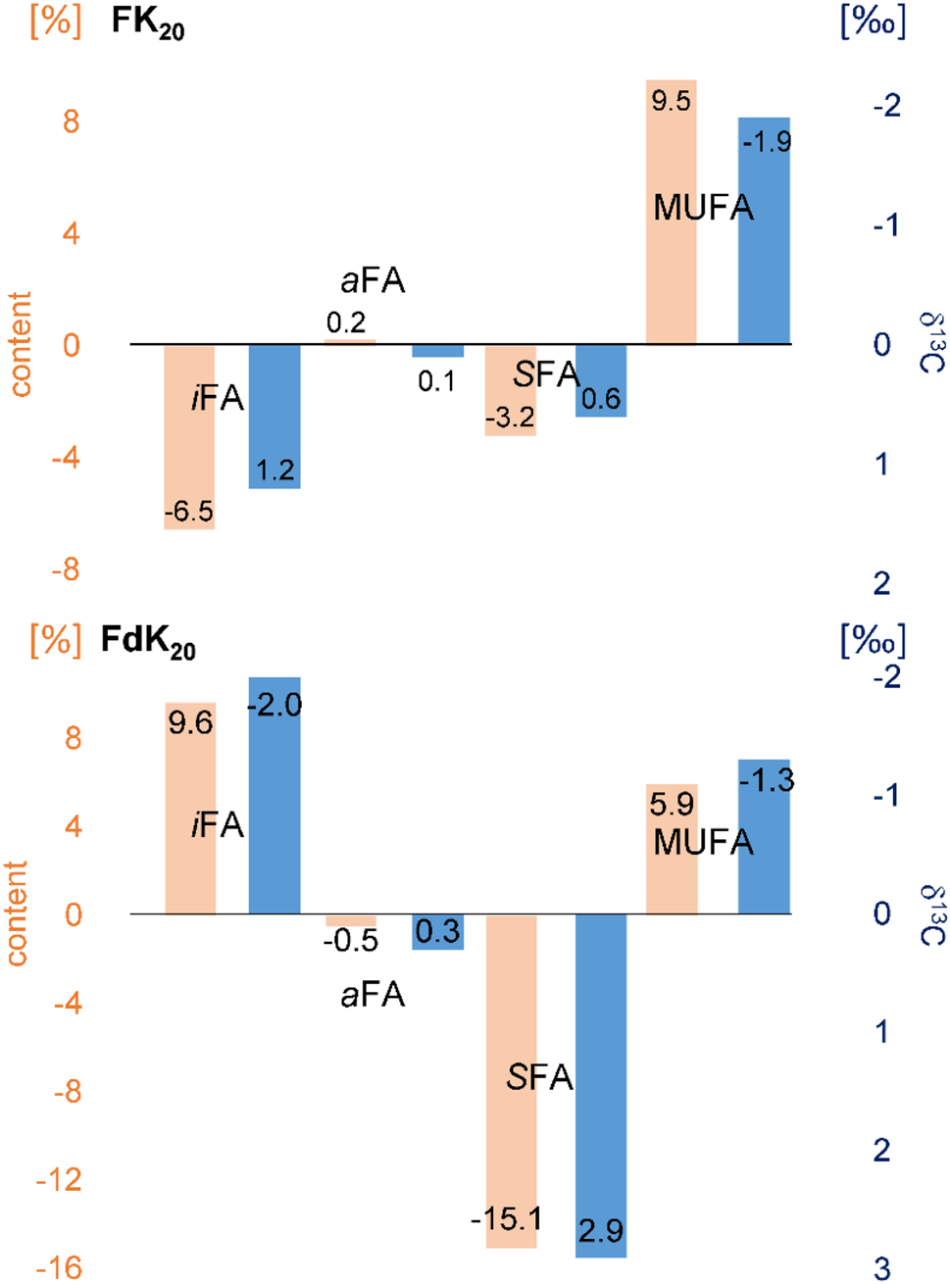
Differences of percentage distribution and δ^13^C values (‰) of the fatty acid groups of the control and the FK_20_- or FdK_20_-treated samples of *Xanthomonas campestris* pathovar (pv) *campestris* (*X. campestris*). The left axis (orange) shows the difference of the percentage distribution of the fatty acid groups between FK_20_ or FdK_20_ – control and the right axis (blue) shows the difference of Σδ^13^C_ind, norm_ values (‰) of the fatty acid groups of FK_20_ or FdK_20_—control.

Treatment with FdK_20_ also strongly changed the FA pattern of *X. campestris* but in opposite direction as FK_20_. Namely, *i*FAs (^C^38.4% → ^FdK^47.8%) and monoenoic FAs were increasing while saturated FAs dropped by ~ 15% (Fig. 2). Still, the direction of changes in the share of *i*FAs was also found to be non-uniform in the case of FK_20_ (“Effect of treatments with the RPM FK_20_ on the fatty acid pattern of the six bacteria samples” section). However, a noticeable exception was observed in the case of *C. michiganensis.* Although FdK_20_ was found to be active (see low MIC value in Table 2) and rich in *i*FAs, no changes were observed in the FA composition. These findings prompted us to determine δ^13^C values (‰) of individual FAs (as methyl esters) with GC-C-IRMS in order to verify potential relationships between growth effects and FA patterns.

### Supporting the findings by compound specific stable carbon isotope analysis (GC-C-IRMS)

Stronger bonds between heavier isotopes^24–26^ indicated that a decrease in the share of a FA was linked with an enrichment in ^13^C (less negative δ^13^C value (‰)). Vice versa, an increase in FA abundance should lead to a depletion in ^13^C (more negative δ^13^C value (‰)). For GC-C-IRMS measurements, FA to be studied must not co-elute with others and the peak area must be within a greater range. Both prerequisites were taken into account by modifying the GC oven program and measuring samples in different dilutions. However, only FAs contributing > 2% to the total fatty acids could be analysed by GC-C-IRMS (“Stable carbon isotope analysis (δ^13^C values (‰)) of FAMEs via gas chromatography-combustion-isotope ratio mass spectrometry (GC-C-IRMS)” section). In dependence of the sample, 75–94% of the bacterial FAs could be studied by GC-C-IRMS (“Stable carbon isotope analysis (δ^13^C values (‰)) of FAMEs via gas chromatography-combustion-isotope ratio mass spectrometry (GC-C-IRMS)” section). Since, a few major fatty acids showed most changes (“Effect of treatments with the RPM FK_20_ on the fatty acid pattern of the six bacteria samples”), the determinations were considered representative. However, in order to make measurements comparable with each other, shares measured by GC-C-IRMS were normalized to 100% in a first step (Eq. 1, “Calculation mode for δ^13^C values (‰) of FAs and groups of FAs via GC-C-IRMS” section). Then, shares of individual FAs (δ^13^C_ind_ values (‰)) were summed up to give the Σδ^13^C_ind_ value (‰) of the sample (Eq. 2). The correctness of both steps was tested with sample *X. perforans* after FdK_20_ treatment in which 94% could be measured by GC-C-IRMS (“94% sample”). Exclusion of several FAs allowed to reduce the considered FA pool to 75% (“75% sample”; only major fatty acids were considered). After normalization to 100% and addition of individual δ^13^C_ind_ values (‰), the “94% sample” showed a Σδ^13^C_ind_ value (‰) of − 26.4‰ compared to − 26.2‰ in the “75% sample”. This small difference verified that the normalization procedure was adequate. Also low standard deviations of < 1%, that means + /- 0.1‰ on the δ scale, showed that the differences were negligible.

GC-C-IRMS evaluation was also based on the four groups of fatty acids (i.e. *i*FAs, *a*FAs, saturated and mono-enoic FAs). In either case, GC-C-IRMS measurements of Σδ^13^C_ind, norm_ values (‰) verified the changes in the FA pattern caused by FK_20_ or FdK_20_ treatments of the bacteria (relative to the control samples) (Table 4, Fig. 2). As predicted, a decrease in the share of a FA group was accompanied with an increase in the ^13^C content which is represented by more positive Σδ^13^C_ind, norm_ values (‰). Last but not least, FA groups that did not change in abundance by the RPM treatment showed no changes in Σδ^13^C_ind, norm_ values (‰).

For example, in *X. perforans* the share of *i*FAs increased from ^C^45% to ^FK^66% and ^FdK^65%, respectively (Table 5). Accordingly, the Σδ^13^C_ind, norm_ value (‰) of *i*FAs (^C^ − 12.0‰) was getting more negative after the treatment (^FK^− 16.6‰ and ^FdK^− 16.5‰) which corresponds with a depletion in ^13^C (Table 5). Vice versa, saturated FAs decreased in relative abundance from ^C^21% to ^FK^18% and ^FdK^6%, in *X. campestris* while the corresponding Σδ^13^C_ind, norm_ values (‰) verified enrichment in ^13^C (^C^ − 4.1‰ to ^FK^ − 3.5‰ or ^FdK^ − 1.2‰) (Table 5). Finally, *A. citrulli* showed negligible changes in the only two FA groups (saturated and monoenoic FAs) and also the Σδ^13^C_ind, norm_ values (‰) of saturated (^C^ − 10.8‰, ^FK^ − 10.6‰ and ^FdK^ − 10.5‰) and monoenoic FAs (^C^ − 11.6‰, ^FK^ − 11.8‰ and ^FdK^ − 11.8‰) remained unchanged (Table 5).

Most remarkably, the reversed change in the *i*FA share of *X. campestris* after treatment with FK_20_ and FdK_20_, respectively, could be verified by GC-C-IRMS (Table 5, Fig. 2). Namely, the increase of *i*FAs in the FdK_20_-treated sample was paired with a depletion in ^13^C (more negative Σδ^13^C_ind, norm_ values (‰)) while FK_20_ caused a decrease of *i*FAs and an increase in the Σδ^13^C_ind, norm_ value (‰) (more positive Σδ^13^C_ind, norm_ values (‰)) (Fig. 2). Although it could not be clarified in this study, the strong changes in the fatty acid composition indicated that the enzyme functionality of fatty acid synthesis was possibly changed by the organisms.

In all experiments, the sum δ^13^C values (‰) of the fatty acids before and after the treatment agreed very well. Within the four groups of fatty acids, changes were most striking in the case of *i*FAs and MUFAs. Smaller changes in the δ^13^C values (‰) were observed for SFA while *a*FAs were barely affected by the treatments. Typically, relevance and δ^13^C values (‰) of *i*FAs and MUFAs changed in opposite direction. Namely, if *i*FAs increased in abundance, the MUFAs content was decreased. This may indicate that the mode of fatty acid synthesis of the organisms was changed due to the impact of RPMs.

## Conclusion

Stable carbon isotope analysis invariably verified the changes in the FA groups of bacteria caused by treatment with FK_20_ and FdK_20_. Also, growth inhibition of bacteria by FK_20_ and FdK_20_ was generally associated with changes in the FA groups except for FdK_20_ in the case of *C. michiganensis* (inhibiting effect but no changes in the FAs). Nevertheless, also this observation could be fully verified by GC-C-IRMS analysis.

For all bacteria that showed an effect, differences were particularly noticeable when *i*FAs were present which is a rather common feature of bacteria. Although each bacterium responded differently and the effects could not be explained mechanistically this time, congruency of changes in FA groups and δ^13^C values (‰) observed in this study may contribute fundamentally to the understanding of the effects of RPMs on bacterial lipid membranes. Similarly, it would be interesting to test if corresponding changes in the fatty acid pattern and stable isotope ratio will also take place in treatments with classic antibiotics such as polymyxin B. In either case the approach used in this study could be helpful in the search not only of novel antimicrobial RPMs but also other antibiotics.

## Supplementary Information

Supplementary Information.

## Data Availability

The datasets used and/or analysed during the current study available from authors upon request.
