# Supplementary material for: Stable isotope analysis confirms substantial changes in the fatty acid composition of bacteria treated with antimicrobial random peptide mixtures (RPMs)
Source: Sci Rep. 2022 Jul 4;12:11230. doi: 10.1038/s41598-022-13134-z (PMC9252987; doi:10.1038/s41598-022-13134-z)
Supplement: Supplementary file 1 — Supplementary Information. [file 41598_2022_13134_MOESM1_ESM.pdf]

Supporting Information for:

Stable Isotope Analysis Confirms Substantial Changes in the Fatty Acid Composition of Bacteria Treated with Antimicrobial Random Peptide Mixtures (RPMs)

Nina Wiedmaier-Czerny[1], Dorothee Schroth[1], Stephanie Krauß[1], Shiri Topman-Rakover[2,3], Aya Brill[2,3], Saul Burdman[3], Zvi Hayouka[2]* and Walter Vetter[1]*

[1] Institute of Food Chemistry, Department of Food Chemistry (170b), University of Hohenheim, D-70593 Stuttgart, Germany

[2] Institute of Biochemistry, Food Science and Nutrition, The Robert H. Smith Faculty of Agriculture, Food and Environment, The Hebrew University of Jerusalem, Rehovot 7610001, Israel

[3] Department of Plant Pathology and Microbiology, Institute of Environmental Sciences, The Robert H. Smith Faculty of Agriculture, Food and Environment, The Hebrew University of Jerusalem, Rehovot 7610001, Israel

* Corresponding authors:

Walter Vetter

Phone: +49 711 459 24016

Fax: +49 711 459 24377

E-Mail: walter.vetter@uni-hohenheim.de

Zvi Hayouka

Phone: +97289489019

Fax: +97289489483

E-Mail: zvi.hayouka@mail.huji.ac.il

Table of contents

1 Figures

2 Tables

3 References

1 Figures

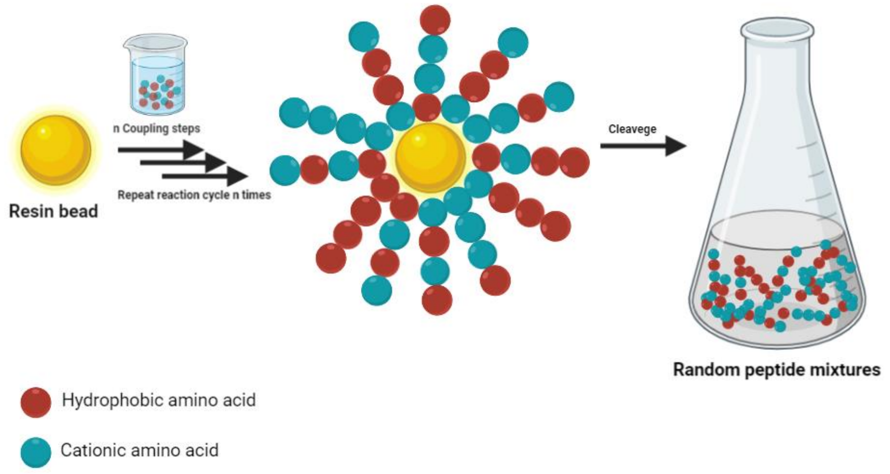

Fig. S1: A schematic representation of the solid synthesis of antimicrobial random peptide mixtures with hydrophobic and cationic amino acids.

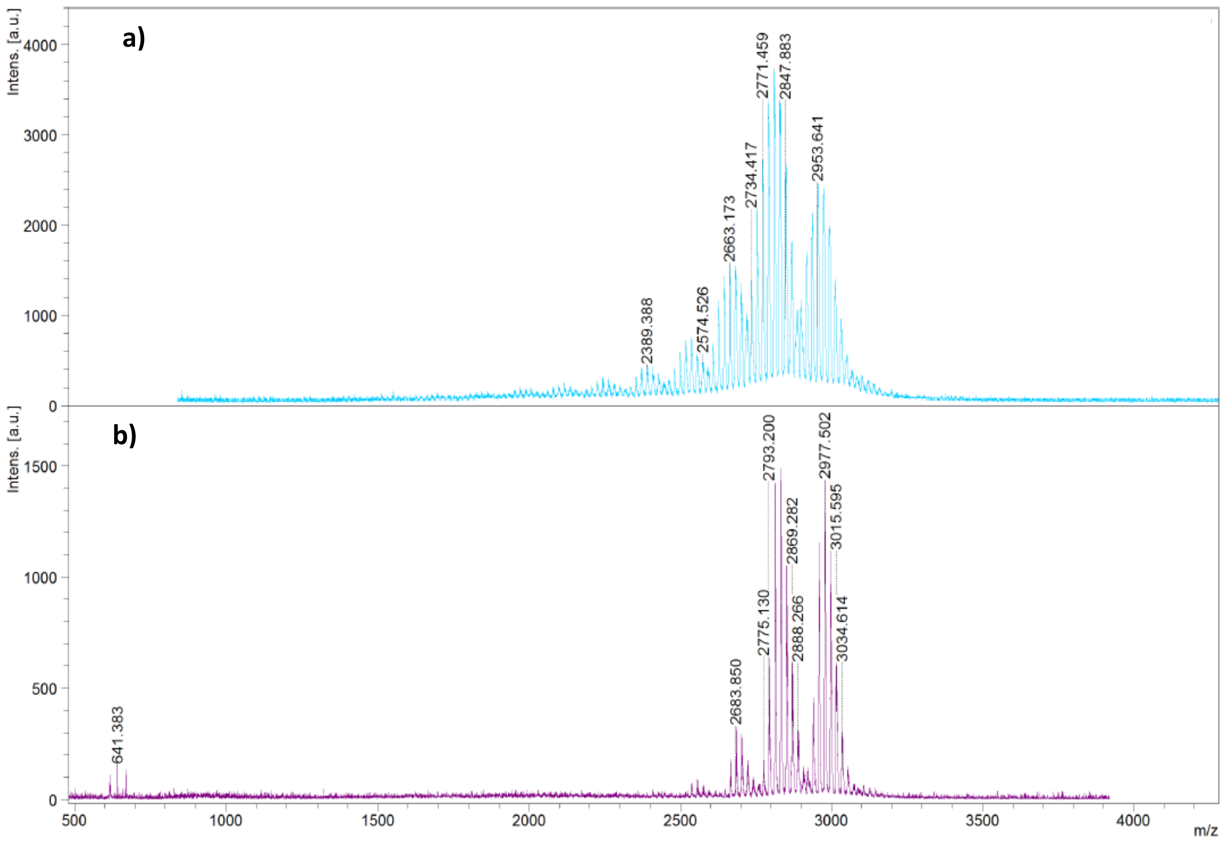

Fig. S2: MALDI-TOF spectrum of RPMs **a)** FK_{20} and **b)** FdK_{20} using the method according to Bauer et al..[1]

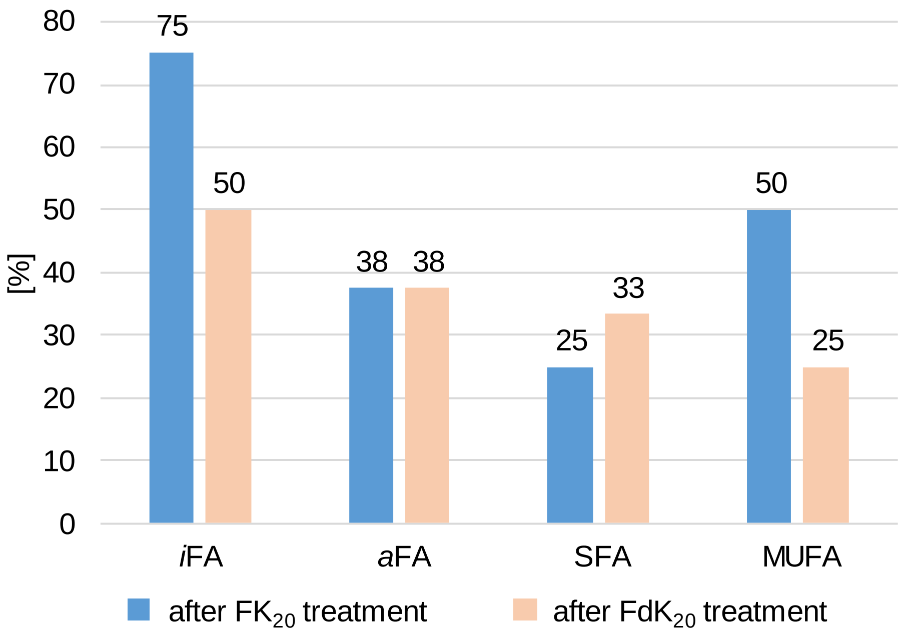

Fig. S3: Percentage distribution of the four fatty acid groups (*i*FA = *iso*-fatty acids, *a*FA = *anteiso*-fatty acids, SFA = saturated fatty acids, MUFA = monounsaturated fatty acids) of all bacteria samples where a significant effect was observed after FK_{20} or FdK_{20} treatment. Values that differed by more than 3% were considered to be an effect.

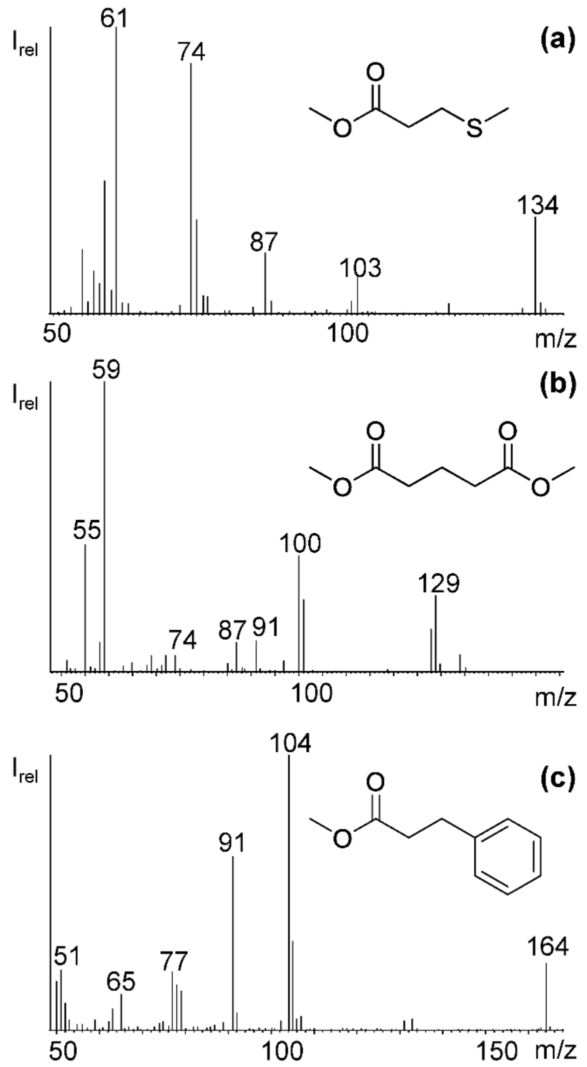

Fig. S4: GC/MS mass spectra of **(a)** 3-(methylthio)propionic acid methyl ester (3-MeS-3:0-ME), **(b)** pentanedioic acid methyl diester (Di5:0-diME), and **(c)** 3-phenylpropanoic acid methyl ester (3-Ph-3:0-ME) with corresponding structures.

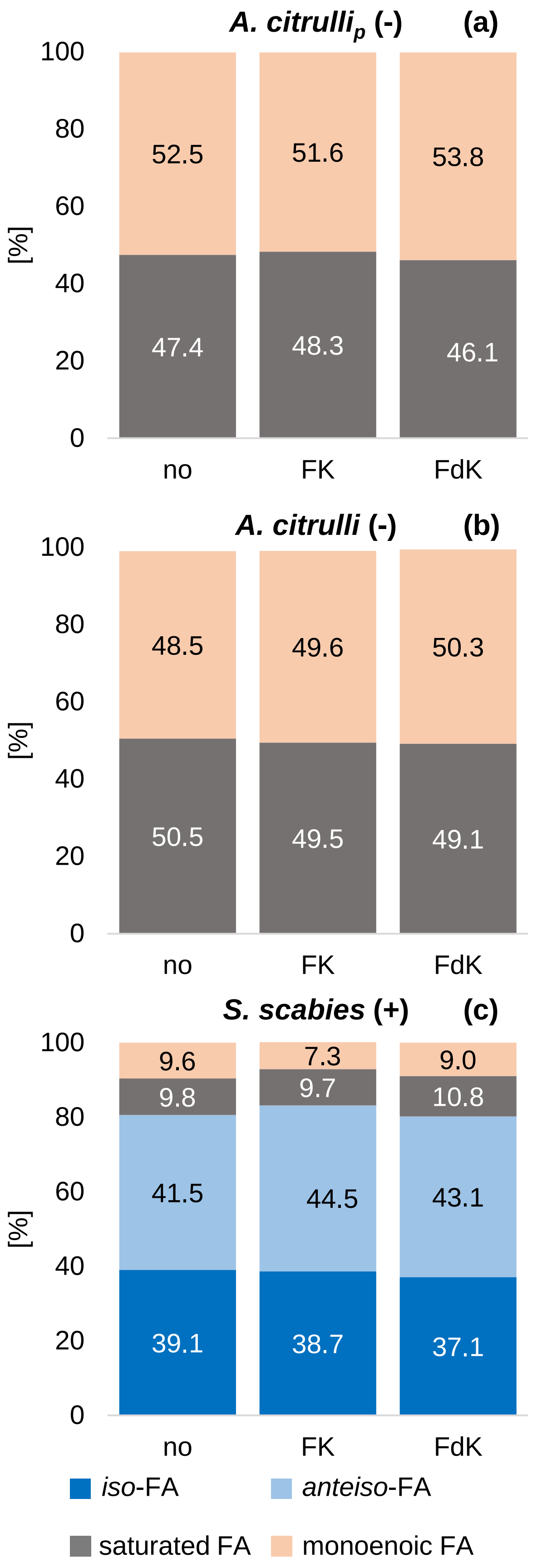

Fig. S5: Percentage distribution of fatty acid groups (Σ*iso*-FAs, Σ*anteiso*-FAs, Σsaturated FAs and Σmonoenoic FAs) of **(a)** *Acidovorax citrulli* (*A. citrulli$_p$*) of the first cultivation half a year before, **(b)** *Acidovorax citrulli* (*A. citrulli*) of the second cultivation, present sample and **(c)** *Streptomyces scabies* (*S. scabies*) samples without treatment and with FK_{20} and FdK_{20} treatment.

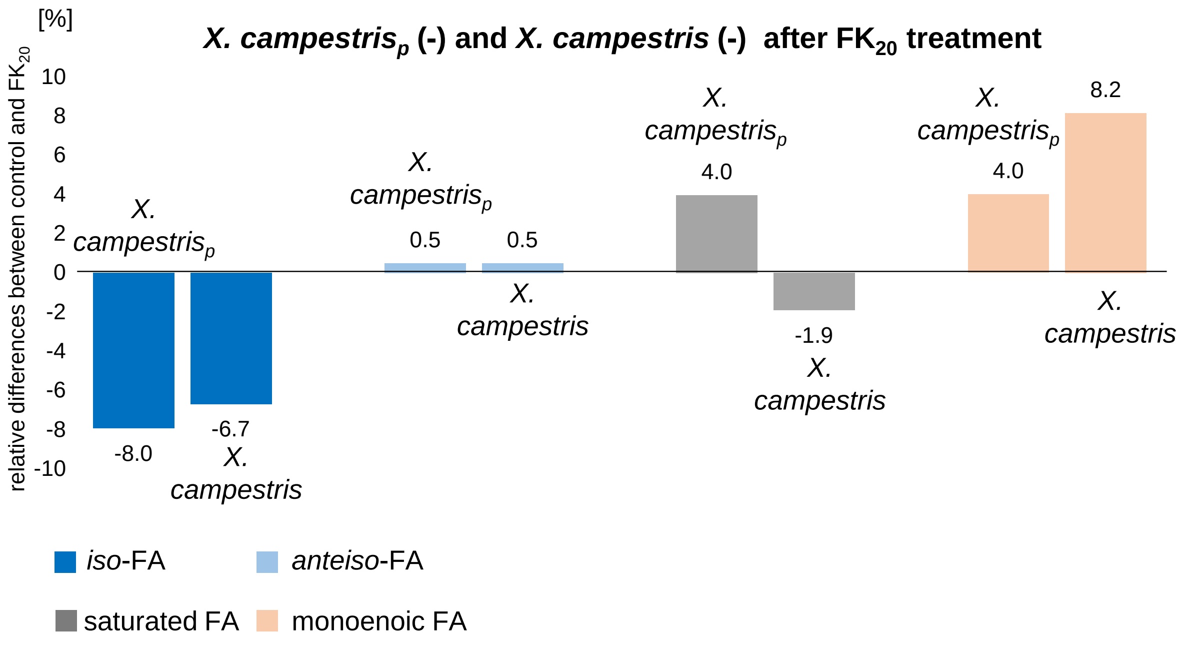

Fig. S6: Relative differences between control and FK_{20} treatment of the four fatty acid groups (*iso*-FAs, *anteiso*-FAs, saturated FAs and monoenoic FAs) of *Xanthomonas campestris* pathovar (pv) *campestris* (*X. campestris*) samples, with *X. campestris$_p$* = sample of the preliminary treatment cultivated half a year before and *X. campestris* = present sample.

2 Tables

Table S1: Calculation of the measurement solutions for the GC-C-IRMS measurements.

		c* [µg/mL]	µL in 100 µL	c** [ng/µL]
C. michiganensis	Control 1	207.3	60	124.4
	Control 2	354.3	40	141.7
	FK_{20} 1	246.7	60	148.0
	FK_{20} 2	274.9	60	164.9
	FdK_{20} 1	404.8	40	161.9
	FdK_{20} 2	367.9	40	147.2
S. scabies	Control 1	208.9	70	146.2
	Control 2	200.0	70	140.0
	FK_{20} 1	930.1	20	186.0
	FK_{20} 2	172.1	70	120.5
	FdK_{20} 1	189.3	70	132.5
	FdK_{20} 2	200.1	70	140.1
P. syringae	Control 1	520.2	30	156.1
	Control 2	458.2	30	137.5
	FK_{20} 1	501.7	30	150.5
	FK_{20} 2	677.7	15	101.7
	FdK_{20} 1	440.9	30	132.3
	FdK_{20} 2	600.2	15	90.0
A. citrulli	Control 1	438.1	20	87.6
	Control 2	377.1	30	113.1
	FK_{20} 1	288.9	30	86.7
	FK_{20} 2	426.3	20	85.3
	FdK_{20} 1	500.1	30	150.0
	FdK_{20} 2	308.5	30	92.6
X. campestris	Control 1	341.4	60	204.8
	Control 2	438.5	60	263.1
	FK_{20} 1	300.5	80	240.4
	FK_{20} 2	260.7	80	208.6
	FdK_{20} 1	401.9	60	241.1
	FdK_{20} 2	428.6	60	257.2
X. perforans	Control 1	302.9	80	242.3
	Control 2	321.9	80	257.5
	FK_{20} 1	309.4	60	185.6
	FK_{20} 2	345.1	60	207.1
	FdK_{20} 1	243.8	100	243.8
	FdK_{20} 2	282.6	100	282.6

* concentration after transesterification

** concentration for measurements with GC-C-IRMS

Table S2: Calculation of the $\delta^{13}C$ values (‰) of all six control plant-pathogenic bacteria after GC-C-IRMS and extrapolation to 100%.

	FA group	FA	A_i	B_i	A_i*B_i	Control $\Sigma\delta^{13}C_{ind, norm}$ [‰]
A. citrulli	SFA	16:0	-22.7	0.427	-9.7	**-10.8**
	MUFA	16:1	-23.4	0.393	-9.2	**-11.6**
		18:1	-15.3	0.076	-1.2	
	sum			0.896		**-22.4**
S. scabies	*i*FA	*i*14:0	-24.5	0.034	-0.8	**-10.2**
		*i*15:0	-23.8	0.079	-1.9	
		*i*16:0	-22.7	0.218	-4.9	
		*i*17:0	-22.4	0.059	-1.3	
	*a*FA	*a*15:0	-24.2	0.257	-6.2	**-11.0**
		*a*17:0	-22.2	0.158	-3.5	
	SFA	16:0	-21.7	0.077	-1.7	**-1.9**
	sum			0.883		**-23.1**
P. syringae	SFA	16:0	-17.3	0.334	-5.8	**-6.4**
	MUFA	16:1	-16.9	0.422	-7.1	**-10.2**
		18:1	-14.1	0.145	-2.0	
	sum			0.901		**-16.6**
C. michiganensis	*i*FA	*i*15:0	-21.9	0.377	-8.3	**-12.3**
		*i*17:0	-26.5	0.049	-1.3	
	*a*FA	*a*15:0	-28.5	0.113	-3.2	**-4.1**
	SFA	14:0	-26.9	0.040	-1.1	**-5.9**
		16:0	-21.8	0.159	-3.5	
	MUFA	17:1	-22.2	0.038	-0.8	**-1.1**
	sum			0.776		**-23.4**
X. campestris	*i*FA	*i*15:0	-21.3	0.237	-5.1	**-8.3**
		*i*16:0	-21.6	0.031	-0.7	
		*i*17:0	-20.1	0.063	-1.3	
	*a*FA	*a*15:0	-21.6	0.166	-3.6	**-4.3**
	SFA	14:0	-25.5	0.018	-0.5	**-4.1**
		15:0	-18.5	0.060	-1.1	
		16:0	-18.2	0.102	-1.8	
	MUFA	16:1	-18.8	0.111	-2.1	**-3.6**
		17:1	-19.4	0.049	-0.9	
	sum			0.836		**-20.3**
X. perforans	*i*FA	*i*13:0	-30.7	0.039	-1.2	**-12.0**
		*i*14:0	-36.5	0.017	-0.6	
		*i*15:0	-25.5	0.191	-4.9	
		*i*16:0	-29.2	0.034	-1.0	
		*i*17:0	-25.7	0.103	-2.7	
	*a*FA	*a*15:0	-25.5	0.112	-2.8	**-3.9**
		*a*17:0	-25.9	0.021	-0.5	
	SFA	14:0	-28.6	0.025	-0.7	**-5.9**
		15:0	-21.5	0.031	-0.7	
		16:0	-24.6	0.148	-3.6	
	MUFA	16:1*n*-7	-20.6	0.138	-2.8	**-3.3**
	sum			0.858		**-25.1**

A_i = $\delta^{13}C_{ind}$ value (‰) of individual FAs
B_i = FA [%]/ 100% = share of each FA
Control $\Sigma\delta^{13}C_{ind, norm}$ [‰] = $\Sigma[A_i*B_i] / (\Sigma B_i)$

Table S3: Calculation of the $\delta^{13}C$ values (‰) of all six **FK$_{20}$** treated plant-pathogenic bacteria after GC-C-IRMS. Extrapolation to 100% and standardization of the treated samples to the sum of the control sample.

	FA group	FA	A_i	B_i	A_i*B_i	FK$_{20}$ $\Sigma\delta^{13}C_{ind}$ [‰]	f [a]	FK$_{20}$ norm.[b]
A. citrulli	SFA	16:0	-22.1	0.418	-9.2	-10.3		**-10.6**
	MUFA	16:1	-22.6	0.403	-9.1	-11.5		**-11.8**
		18:1	-15.2	0.075	-1.1			
	sum			0.896		-21.8	1.03	**-22.4**
S. scabies	*i*FA	*i*14:0	-25.7	0.032	-0.8	-10.0		**-9.8**
		*i*15:0	-23.7	0.083	-1.9			
		*i*16:0	-23.1	0.204	-4.7			
		*i*17:0	-23.4	0.068	-1.6			
	*a*FA	*a*15:0	-24.4	0.271	-6.6	-11.7		**-11.4**
		*a*17:0	-22.8	0.174	-4.0			
	SFA	16:0	-22.7	0.076	-1.7	-1.9		**-1.9**
	sum			0.907		-23.6	0.98	**-23.1**
P. syringae	SFA	16:0	-18.8	0.319	-6.0	-6.5		**-6.1**
	MUFA	16:1	-18.2	0.435	-7.9	-11.3		**-10.5**
		18:1	-15.1	0.168	-2.5			
	sum			0.922		-17.8	0.93	**-16.6**
C. michiganensis	*i*FA	*i*15:0	-23.5	0.330	-7.8	-12.1		**-11.5**
		*i*17:0	-28.2	0.045	-1.3			
	*a*FA	*a*15:0	-29.6	0.111	-3.3	-4.4		**-4.2**
	SFA	14:0	-26.7	0.043	-1.1	-6.9		**-6.5**
		16:0	-23.5	0.170	-4.0			
	MUFA	17:1	-22.3	0.045	-1.0	-1.4		**-1.3**
	sum			0.745		-24.8	0.94	**-23.4**
X. campestris	*i*FA	*i*15:0	-23.0	0.188	-4.3	-7.5		**-7.1**
		*i*16:0	-21.7	0.031	-0.7			
		*i*17:0	-22.0	0.061	-1.3			
	*a*FA	*a*15:0	-21.9	0.171	-3.7	-4.4		**-4.2**
	SFA	14:0	-21.5	0.023	-0.5	-3.7		**-3.5**
		15:0	-18.7	0.034	-0.6			
		16:0	-20.5	0.099	-2.0			
	MUFA	16:1	-19.9	0.207	-4.1	-5.8		**-5.5**
		17:1	-21.4	0.036	-0.8			
	sum			0.849		-21.3	0.95	**-20.3**
X. perforans	*i*FA	*i*13:0	-22.1	0.078	-1.7	-15.9		**-16.6**
		*i*14:0	-29.9	0.040	-1.2			
		*i*15:0	-23.5	0.272	-6.4			
		*i*16:0	-27.4	0.050	-1.4			
		*i*17:0	-23.6	0.166	-3.9			
	*a*FA	*a*15:0	-25.8	0.076	-2.0	-2.8		**-2.9**
		*a*17:0	-26.3	0.023	-0.6			
	SFA	14:0	-28.7	0.020	-0.6	-3.7		**-3.8**
		15:0	-22.4	0.023	-0.5			
		16:0	-23.2	0.097	-2.3			
	MUFA	16:1*n-7*	-20.5	0.063	-1.3	-1.7		**-1.8**
		16:1*n-9*	-27.6	0.009	-0.2			
	sum			0.917		-24.1	1.05	**-25.1**

A_i = $\delta^{13}C_{ind}$ value (‰) of individual FAs

B_i = FA [%]/ 100% = share of each FA

FK$_{20}$ $\Sigma\delta^{13}C_{ind}$ [‰] = $\Sigma[A_i*B_i]$ / (ΣB_i)

[a] $f = \Sigma\delta^{13}C_{cont} / \Sigma\delta^{13}C_{treat}$

[b] normalized $\Sigma\delta^{13}C$ values ($\Sigma\delta^{13}C_{ind, norm}$) = $\Sigma\delta^{13}C_{ind}$ (FA group) * f

Table S4: Calculation of the $\delta^{13}C$ values (‰) of all six **FdK20** treated plant-pathogenic bacteria after GC-C-IRMS. Extrapolation to 100% and standardization of the treated samples to the sum of the control sample.

	FA group	FA	A_i	B_i	$A_i{*}B_i$	FdK20 $\Sigma\delta^{13}C_{ind}$ [‰]	f^a	FdK20 norm.[b]
A. citrulli	SFA	16:0	-22.7	0.423	-9.6	-10.6		**-10.6**
	MUFA	16:1	-23.0	0.405	-9.3	-11.9		**-11.8**
		18:1	-18.5	0.078	-1.4			
	sum			0.907		-22.5	0.98	**-22.4**
S. scabies	*i*FA	*i*14:0	-26.9	0.034	-0.9	-9.8		**-9.8**
		*i*15:0	-23.9	0.075	-1.8			
		*i*16:0	-22.8	0.211	-4.8			
		*i*17:0	-23.3	0.051	-1.2			
	*a*FA	*a*15:0	-23.7	0.276	-6.5	-11.4		**-11.3**
		*a*17:0	-22.5	0.156	-3.5			
	SFA	16:0	-22.2	0.080	-1.8	-2.0		**-2.0**
	sum			0.881		-23.3	0.99	**-23.1**
P. syringae	SFA	16:0	-19.2	0.338	-6.5	-7.0		**-6.4**
	MUFA	16:1	-18.9	0.426	-8.0	-11.2		**-10.2**
		18:1	-14.5	0.158	-2.3			
	sum			0.922		-18.2	0.91	**-16.6**
C. michiganensis	*i*FA	*i*15:0	-21.5	0.395	-8.5	-11.9		**-12.8**
		*i*17:0	-21.8	0.052	-1.1			
	*a*FA	*a*15:0	-24.2	0.114	-2.8	-3.4		**-3.7**
	SFA	14:0	-21.9	0.038	-0.8	-5.5		**-5.9**
		15:0	-26.3	0.014	-0.4			
		16:0	-21.0	0.156	-3.3			
	MUFA	17:1	-20.3	0.040	-0.8	-1.0		**-1.1**
	sum			0.808		-21.8	1.07	**-23.4**
X. campestris	*i*FA	*i*15:0	-21.1	0.305	-6.4	-10.1		**-10.3**
		*i*16:0	-20.8	0.035	-0.7			
		*i*17:0	-19.0	0.094	-1.8			
	*a*FA	*a*15:0	-20.4	0.171	-3.5	-3.9		**-4.0**
	SFA	14:0	-20.5	0.008	-0.2	-1.2		**-1.2**
		15:0	-17.5	0.021	-0.4			
		16:0	-19.8	0.028	-0.6			
	MUFA	16:1	-18.9	0.109	-2.1	-4.8		**-4.9**
		17:1	-19.5	0.112	-2.2			
	sum			0.882		-20.1	1.01	**-20.3**
X. perforans	*i*FA	*i*13:0	-26.8	0.090	-2.4	-17.4		**-16.5**
		*i*14:0	-33.3	0.033	-1.1			
		*i*15:0	-25.7	0.247	-6.4			
		*i*16:0	-30.8	0.046	-1.4			
		*i*17:0	-25.6	0.200	-5.1			
	*a*FA	*a*15:0	-27.3	0.065	-1.8	-2.7		**-2.6**
		*a*17:0	-27.6	0.028	-0.8			
	SFA	14:0	-27.7	0.024	-0.7	-5.1		**-4.8**
		15:0	-24.7	0.010	-0.2			
		16:0	-27.1	0.144	-3.9			
	MUFA	16:1*n-7*	-19.9	0.052	-1.0	-1.3		**-1.2**
		16:1*n-9*	-30.3	0.006	-0.2			
	sum			0.943		-26.5	0.95	**-25.1**

$A_i = \delta^{13}C_{ind}$ value (‰) of individual FAs
B_i = FA [%]/ 100% = share of each FA
FdK20 $\Sigma\delta^{13}C_{ind}$ [‰] = $\Sigma[A_i{*}B_i] / (\Sigma B_i)$
[a] $f = \Sigma\delta^{13}C_{cont} / \Sigma\delta^{13}C_{treat}$
[b] normalized $\Sigma\delta^{13}C$ values ($\Sigma\delta^{13}C_{ind,\,norm}$) = $\Sigma\delta^{13}C_{ind}$ (FA group) * f

Table S5: Average of percentage composition of the FAs of control sample and FK_{20} and FdK_{20} treated sample of *Streptomyces scabies* (*S. scabies*) (n = 2). Comparison of the present sample and of pathogenic and scab-suppressive *S. scabies* analysed by Ndowora et al.[2] and of two different groups of *S. scabies* analysed by Paradis et al.[3].

FAME	*S. scabies* control [%]	*S. scabies* FK_{20} [%]	*S. scabies* FdK_{20} [%]	*S. scabies* pathogenic/ scab-suppressive by Ndowora et al. (1996)[2] [%]	*S. scabies* group 1/ group 2 A by Paradis et al. (1994)[3] [%]
variety	18	18	18	16	13
*i*13:0				- / 0.3	1.33/ < 1
*i*14:0	3.4	3.1	3.4	11.0/ 7.6	3.13/ 2.17
14:0	0.2	0.2	0.2		4.59/ 1.03
*i*15:0	8.1	8.4	7.6	9.2/ 13.5	11.20/ 17.46
*a*15:0	26.1	27.5	27.9	11.0/ 21.5	15.30/ 23.77
15:0	1.2	1.3	1.7	5.3/ 3.9	2.55/ 1.31
*i*16:0	22.2	20.6	21.4	27.6/ 25.3	7.33/ 10.79
16:0	7.9	7.7	8.1	5.1/ 2.3	30.78/ 15.01
*i*17:0	6.0	6.9	5.1	2.0/ 2.1	2.68/ 8.18
*a*17:0	16.0	17.6	15.8	4.6/ 6.5	4.58/ 10.80
17:0	0.5	0.7	1.0	0.5/ -	
*i*18:0	0.1	0.1	0.1		
15:1				0.8/ 0.9	
*i*16:1				7.3/ 3.2	
16:1*n-7* (#2)	0.6	0.4	2.5	6.9/ 4.2	12.28/ 3.64
9-methyl-16:0				3.7/ 3.4	
*i*17:1 (#2)	1.4	1.0	0.6		1.63/ 2.66
*a*17:1 (#3)	1.4	1.1	1.1	2.3/ 3.8	< 1/ 1.57
17:1 (#4)	0.9	0.7	1.5		
17:1 (#5)	3.4	2.4	1.5		
17:1*n-8* (#6)				1.5/ 0.7	
17:1 (#8)	0.3	0.3	0.3		
18:1 (#1)	1.8	1.6	1.6		
Instrument	GC/MS			GC/FID	GC/FID
Column	60 m x 0.25 mm i.d. 90% biscyanopropyl, 10% cyanopropylphenyl polysiloxane capillary column			25 m x 0.2 mm i.d. 5% phenyl methyl silicone fused silica capillary column	25 m x 0.2 mm i.d. fused silica column

Table S6: Average of percentage composition of the FAs of control sample and FK_{20} and FdK_{20} treated sample of *Acidovorax citrulli* (*A. citrulli*) (n = 2). Comparison of the present sample (*A. citrulli*), the sample cultivated half a year ago (*A. citrulli$_p$*) and exemplarily by means of two different haplotypes (A/ E) of *A. avenae* subsp*. citrulli*, which were analysed by Walcott et al..[4]

FAME	*A. citrulli$_p$* control [%]	*A. citrulli$_p$* FK_{20} [%]	*A. citrulli$_p$* FdK_{20} [%]	*A. citrulli* control [%]	*A. citrulli* FK_{20} [%]	*A. citrulli* FdK_{20} [%]	*A. citrulli* haplotype A/ E by Walcott et al. (1999)[4] [%]
variety	20	19	19	16	16	18	13
10:0				0.1	0.1	0.1	0.8/ 0.7
12:0	0.2	0.2	0.2	2.9	3.0	2.5	3.0/ 3.6
13:0						0.01	1.7/ 1.8
14:0	2.2	1.9	1.8	3.2	3.2	2.8	
*i*15:0				0.01	0.02	0.01	
*a*15:0	0.01			0.01	0.01		
15:0	0.4	0.6	0.9	1.1	1.0	1.0	2.0/ 1.7
*i*16:0				0.02	0.02		
16:0	44.1	44.7	42.5	42.7	41.8	42.3	31.3/ 28.4
17:0	0.2	0.2	0.3	0.2	0.2	0.2	0.3/ 2.2
18:0	0.3	0.6	0.4	0.3	0.2	0.3	
14:1	0.04	0.02	0.03			0.03	
monoenoic						0.04	
14:1*n-5* (#2)	0.09	0.06	0.06				
15:1*n-5* (#2)	0.2	0.2	0.2				- / 0.6
16:1 (#1)	1.4	1.0	1.4	1.3	1.5	1.4	
16:1*n-7* (#2)	42.5	41.6	43.7	39.3	40.3	40.5	43.7/ 41.5
17:1 (#4)	0.06	0.09	0.03				
17:1 (#5)	0.05	0.05	0.02				
17:1*n-8* (#6)	0.02	0.02	0.02				
17:1 (#7)	0.01	0.02	0.01				
monoenoic						0.2	
18:1 (#3)	0.2	0.2	0.2	0.2	0.2	0.2	
18:1*n-9* (#4)	0.1	0.1	0.1	0.1	0.1	0.1	
18:1*n-7* (#5)	7.82	8.2	7.9	7.6	7.5	7.8	8.0/ 5.4
18:1 (#6)	0.01	0.01	0.01				
3-OH-10:0 (#1)	0.5	0.3	0.4	1.0	0.9	0.5	6.7/ 8.4
3-OH-11:0							0.3/ 0.4
3-OH-12:1							2.4/ 4.2
3-OH-12:0 (#3)							1.0/ 3.2
Instrument	GC/MS						Gas-liquid chromatography
Column	60 m x 0.25 mm i.d. 90% biscyanopropyl, 10% cyanopropylphenyl polysiloxane capillary column						30 m x 0.25 mm i.d. phenyl methyl silicone fused silica capillary column

Table S7: Average of percentage composition of the FAs of control sample and FK_{20} and FdK_{20} treated sample of *Xanthomonas campestris* pathovar (pv) *campestris* (*X. campestris*) (n = 2). Comparison of the present sample (*X. campestris*), the sample cultivated half a year ago (*X. campestris$_p$*) and of *X. campestris* sample analysed by Vauterin et al..[5]

FAME	*X. campestris$_p$* control [%]	*X. campestris$_p$* FK_{20} [%]	*X. campestris$_p$* FdK_{20} [%]	*X. campestris* control [%]	*X. campestris* FK_{20} [%]	*X. campestris* FdK_{20} [%]	*X. campestris* by Vauterin et al. (1966)[5] [%]
variety	32	33	32	29	29	25	26
10:0	0.02	0.04	0.04	0.7	1.1	0.3	0.6 (± 0.3)
*i*11:0	0.4	0.3	0.3	4.2	2.8	3.6	4.5 (± 0.7)
*a*11:0	0.04	0.02	0.02	0.4	0.2	0.2	
*i*12:0	0.02	0.01	0.01				
12:0	0.03	0.06	0.02	0.1	0.3		
*i*13:0	1.0	0.4	0.7	0.3	0.2	0.2	0 (± 0.3)
*a*13:0	0.2	0.1	0.2	0.1	0.1	0.1	
13:0	0.1	0.1	0.1	0.1	0.04	0.02	
*i*14:0	1.1	0.7	1.0	0.8	0.7	0.7	0.7 (± 0.5)
14:0	2.1	2.1	1.8	1.8	2.3	0.8	0.8 (± 0.4)
*i*15:0	34.5	24.6	36.2	23.7	18.8	30.5	26.5 (± 3.4)
*a*15:0	19.5	19.5	20.8	16.6	17.1	17.1	13.9 (± 2.2)
15:0	6.0	6.3	4.6	6.1	3.4	2.1	1.2 (± 0.6)
*i*16:0	2.2	2.7	2.6	3.1	3.1	3.5	3.2 (± 1.3)
16:0	7.2	10.5	5.9	10.2	9.9	2.8	3.6 (± 1.0)
*i*17:0	3.9	6.3	5.0	6.3	6.1	9.4	6.8 (± 1.4)
*a*17:0	0.5	1.1	0.6	0.8	1.0	1.4	0.8 (± 0.5)
17:0	0.4	0.7	0.4	0.9	0.2		
18:0	0.6	0.5	0.09	0.1	0.7	0.1	
14:1	0.04	0.05	0.05				
14:1	0.02	0.02	0.02				
*i*15:1							0.4 (± 0.4)
*a*15:1 (#1)	0.7	0.3	0.5	0.6	0.2	0.4	
15:1	0.1	0.1	0.1				0.6 (± 0.4)
16:1*n-9* (#1)	1.2	1.9	1.2	2.0	2.3	0.9	0.9 (± 0.7)
16:1*n-7* (#2)	11.2	13.9	12.3	11.1	20.7	10.9	12.7 (± 2.0)
i17:1 (#2)						0.3	
a17:1 (#3)	4.7	4.0	3.2	4.9	3.6	11.2	
17:1*n-8* (#6)	1.7	2.4	1.4	3.3	1.7	1.5	1.4 (± 0.5)
17:1 (#7)	0.1	0.2	0.1	0.2	0.3	0.3	
18:1*n-9* (#4)		0.2		0.7	1.7		0.2 (± 0.3)
18:1*n-7* (#5)	0.04	0.3	0.09	0.5	0.7		
3-OH-10:0 (#1)							0.0 (± 0.1)
3-OH-*i*11:0 (#2)	0.2	0.2	0.1	0.5	0.4	0.9	2.8 (± 0.4)
3-OH-11:0							0.1 (± 0.2)
3-OH-*i*12:0							0.2 (± 0.3)
3-OH-12:0 (#3)	0.05	0.1	0.1	0.1	0.2		2.6 (± 0.5)
3-OH-*i*13:0 (#4)	0.3	0.2	0.2	0.1	0.1	0.2	4.7 (± 0.7)
3-OH-13:0							0.3 (± 0.3)
3-OH-*i*17:0							0.1 (± 0.2)
Instrument	GC/MS						Gas-liquid chromatography
column	60 m x 0.25 mm i.d. 90% biscyanopropyl, 10% cyanopropylphenyl polysiloxane capillary column						25 m x 0.2 mm i.d. methyl phenyl silicone fused silica capillary column

Table S8: Average of percentage composition of the FAs of control sample and FK_{20} and FdK_{20} treated sample of *Xanthomonas perforans* (*X. perforans*) (n = 2) of the present study.

	X. perforans control [%]	*X. perforans* FK_{20} [%]	*X. perforans* FdK_{20} [%]
variety	29	29	26
10:0	0.5	0.3	0.2
*i*11:0	2.4	1.2	0.5
*a*11:0	0.2	0.1	
*i*12:0	0.5	0.7	0.6
12:0	0.4	0.2	0.2
*i*13:0	3.9	7.8	9.0
*a*13:0	1.1	1.0	1.0
13:0	0.1	0.1	0.04
*i*14:0	1.7	4.0	3.3
14:0	2.5	2.0	2.4
*i*15:0	19.1	27.2	24.7
*a*15:0	11.2	7.6	6.5
15:0	3.1	2.3	1.0
*i*16:0	3.4	5.0	4.6
16:0	14.8	9.7	14.4
*i*17:0	10.3	16.6	20.0
*a*17:0	2.1	2.3	2.8
17:0	1.5	0.7	0.6
18:0	0.4	0.4	0.4
16:1 (#1)	1.4	0.9	0.6
16:1*n-7* (#2)	13.8	6.3	5.2
17:1 (#1)		0.8	0.3
*i*17:1 (#2)	1.7	0.9	0.5
*a*17:1 (#3)		0.4	
17:1*n-8* (#6)	1.4	0.5	0.3
17:1 (#7)	0.3	0.2	0.2
18:1*n-9* (#4)	0.8	0.3	0.4
18:1*n-7* (#5)	0.7	0.3	0.4
3-OH-*i*11:0 (#2)	0.5	0.4	
3-OH-12:0 (#3)	0.2		
3-OH-*i*13:0 (#4)	0.2		
Instrument	GC/MS		
Column	60 m x 0.25 mm i.d. 90% biscyanopropyl, 10% cyanopropylphenyl polysiloxane capillary column		

Table S9: Average of percentage composition of the fatty acids of control sample and FK_{20} and FdK_{20} treated sample of *Clavibacter michiganensis* (*C. michiganensis*) (n = 2). Comparison of the present sample and of 45 reference strains analysed by Gitaitis and Beaver.[6]

FAME	*C. michiganensis* control [%]	*C. michiganensis* FK_{20} [%]	*C. michiganensis* FdK_{20} [%]	*C. michiganensis* spp. of 45 reference strains[6] [%]
variety	31	31	31	7
10:0	0.7	1.0	0.6	
*i*11:0	5.4	6.0	4.5	
*a*11:0	0.1	0.2	0.1	
12:0				1.8 (± 3.2)
saturated	0.1	0.1	0.1	
*i*13:0	0.4	0.4	0.5	
*a*13:0	0.1	0.1	0.1	
13:0	0.02	0.01	0.01	
*i*14:0	0.7	0.6	0.7	
14:0	4.0	4.3	3.8	
*i*15:0	37.8	33.1	39.5	0.9 (± 1.2)
*a*15:0	11.3	11.2	11.2	40.9 (± 13.8)
15:0	1.7	1.5	1.3	
*i*16:0	1.2	1.1	1.1	13.9 (± 7.0)
16:0	16.0	17.1	15.8	3.7 (± 3.5)
*i*17:0	5.0	4.6	5.3	
*a*17:0	0.4	0.4	0.4	21.3 (± 10.0)
17:0	0.3	0.3	0.3	
18:0	0.3	0.4	0.3	
*i*19:0	0.2	0.2	0.3	
*a*15:1 (#1)	1.0	1.2	0.9	8.5 (± 13.2)
16:1 (#1)	2.8	3.7	2.5	
16:1*n-7* (#2)	1.4	1.9	1.6	
*a*17:1 (#3)	2.2	2.5	2.3	
17:1 (#5)	3.8	4.6	4.1	
17:1 (#8)	1.3	1.4	1.2	
18:1*n-9* (#4)	0.8	1.0	0.9	
18:1*n-7* (#5)	0.3	0.4	0.4	
monoenoic (#1)	0.2	0.2	0.2	
monoenoic (#2)	0.2	0.2	0.2	
3-OH-*i*11:0 (#2)	0.5	0.8	0.3	
3-OH-*i*13:0 (#4)	0.1	0.2	0.1	
Instrument	GC/MS			Gas-liquid chromatography
Column	60 m x 0.25 mm i.d. 90% biscyanopropyl, 10% cyanopropylphenyl polysiloxane capillary column			30 m x 0.25 mm i.d. phenyl methyl silicone fused silica capillary column

Table S10: Average of percentage composition of the FAs of control sample and FK_{20} and FdK_{20} treated sample of *Pseudomonas syringae* pv. *tomato* (*P. syringae*) (n = 2). Comparison of the present sample and a *P. syringae* sample analysed by Stead.[7]

	P. syringae control [%]	*P. syringae* FK_{20} [%]	*P. syringae* FdK_{20} [%]	*P. syringae* sample by Stead[7] [%]
variety	14	13	11	10
10:0	0.1			trace
12:0	5.1	3.9	4.0	4.7 (± 0.3)
*a*13:0	0.03	0.03		
14:0	0.4	0.2	0.3	0.2 (± 0.1)
*i*16:0	0.02	0.04	0.05	
16:0	33.8	32.1	34.0	26.0 (± 1.4)
*i*17:0				trace
17:0	0.1	0.2	0.2	
18:0	2.0	2.2	2.0	
16:1*n-7* (#2)	42.6	43.8	42.9	40.5 (± 1.8)
18:1 (#2)	1.0	0.2	0.3	
18:1*n-9* (#4)	0.2	0.2		
18:1*n-7* (#5)	14.6	16.9	16.0	17.8 (± 1.1)
3-OH-10:0 (#1)	1.1	0.8	0.9	3.0 (± 0.4)
2-OH-12:0	0.2	0.2	0.2	2.6 (± 0.1)
3-OH-12:0 (#3)				4.0 (± 0.2)
Instrument	GC/MS			GC/FID
Column	60 m x 0.25 mm i.d. 90% biscyanopropyl, 10% cyanopropylphenyl polysiloxane capillary column			25 m methyl silicone fused silica capillary column

Table S11: Bulk $\delta^{13}C$ values of *X. campestris*$_p$, *X. campestris* and *X. perforans* measured with EA-IRMS and calculated after GC-C-IRMS measurements of the individual FAs. Bulk $\delta^{13}C$ values of *X. campestris*$_p$ and *X. campestris* (control and FdK_{20}) of the lyophilisate.

		**$\delta^{13}C$ [‰] (EA-IRMS)**	**standard deviation**	**FK_{20}-control or FdK_{20}-control**	**$\delta^{13}C$ [‰] (GC-C-IRMS)**	**FK_{20}-control or FdK_{20}-control**
FAME	*X. campestris* Control	-22.3	0.26		-20.4	
	X. campestris FK_{20}	-22.4	0.07	-0.11	-21.3	-0.97
	X. campestris FdK_{20}	-20.4	0.01	1.87	-20.1	0.22
	X. campestris$_p$ FK_{20}	-21.7	0.08	0.75		
	X. campestris$_p$ FdK_{20}	-21.3	0.19	0.41		
	X. campestris$_p$ FK_{20}	-21.7	0.08	0.75		
Lyophilisate	*X. campestris*$_p$ Control	-19.3	0.20			
	X. campestris$_p$ FdK_{20}	-19.2	0.05	0.03		
	X. campestris Control	-19.9	0.02			
	X. campestris FdK_{20}	-19.3	0.14	0.61		
FAME	*X. perforans* Control	-24.3	0.35		-25.1	
	X. perforans FK_{20}	-24.2	0.22	0.07	-24.0	1.10
	X. perforans FdK_{20}	-26.3	0.03	-2.04	-26.4	-1.29

3 References

[1] Bauer, T.S., Menagen, B., Avnir, D., Hayouka, Z. Random peptide mixtures entrapped within a copper-cuprite matrix: new antimicrobial agent against methicillin-resistant *Staphylococcus aureus*. *Scientific Reports* **9.1**, 1-8; https://doi.org/10.1038/s41598-019-47315-0 (2019).

[2] Ndowora, T.C.R., Kinkel, L.L., Jones, R.K., Anderson, N.A. Fatty acid analysis of pathogenic and suppressive strains of *Streptomyces* species isolated in Minnesota. *Phytopathology* **86.2**, 138–143; DOI: 10.1094/Phyto-86-138 (1996).

[3] Paradis, E. *et al.* Fatty acid and protein profiles of *Streptomyces scabies* strains isolated in eastern Canada. *International Journal of Systematic and Evolutionary Microbiology* **44.3**, 561-564; https://doi.org/10.1099/00207713-44-3-561 (1994).

[4] Walcott, R.R., Langston, D.B., Sanders, F.H., Gitaitis, R.D. Investigating intraspecific variation of *Acidovorax avenae* subsp. *citrulli* using DNA fingerprinting and whole cell fatty acid analysis. *Phytopathology* **90.2**, 191–196; https://doi.org/10.1094/PHYTO.2000.90.2.191 (2000).

[5] Vauterin, L., Yang, P., Swings, J. Utilization of fatty acid methyl esters for the differentiation of new *Xanthomonas* species. *International Journal of Systematic and Evolutionary Microbiology* **46.1**, 298-304; https://doi.org/10.1099/00207713-46-1-298 (1996).

[6] Gitaitis, R.D., Beaver, R.W. Characterization of fatty acid methyl easter content of *Clavibacter michiganensis* subsp. *michiganensis*. *Phytopathology* **80.4**, 318-321; (1990).

[7] Stead, D.E. Grouping of plant-pathogenic and some other Pseudomonas spp. by using cellular fatty acid profiles. *International Journal of Systematic and Evolutionary Microbiology* **42.2**, 281-295. https://doi.org/10.1099/00207713-42-2-281 (1992).
